# Combined the SMAC mimetic and BCL2 inhibitor sensitizes neoadjuvant chemotherapy by targeting necrosome complexes in tyrosine aminoacyl-tRNA synthase-positive breast cancer

**DOI:** 10.1186/s13058-020-01367-7

**Published:** 2020-11-25

**Authors:** Kyung-Min Lee, Hyebin Lee, Dohyun Han, Woo Kyung Moon, Kwangsoo Kim, Hyeon Jeong Oh, Jinwoo Choi, Eun Hye Hwang, Seong Eun Kang, Seock-Ah Im, Kyung-Hun Lee, Han Suk Ryu

**Affiliations:** 1 Center for Medical Innovation, Biomedical Research Institute, Seoul National University Hospital, Seoul, South Korea; 2 Department of Radiation Oncology, Kangbuk Samsung Hospital, Sungkyunkwan University School of Medicine, Seoul, South Korea; 3 Proteomics Core Facility, Biomedical Research Institute, Seoul National University Hospital, Seoul, South Korea; 4 Department of Radiology, Seoul National University Hospital, Seoul National University College of Medicine, Seoul, South Korea; 5 Division of Clinical Bioinformatics, Biomedical Research Institute, Seoul National University Hospital, Seoul, South Korea; 6 Department of Pathology, National Cancer Center, Goyang-si, South Korea; 7 Department of Industrial Engineering Seoul National University, Seoul, South Korea; 8 Department of Pathology, Seoul National University Hospital, Seoul National University College of Medicine, Seoul, South Korea; 9 Department of Internal Medicine, Seoul National University Hospital, Seoul National University College of Medicine, Seoul, South Korea

**Keywords:** Breast cancer, SMAC mimetic, BCL2 inhibitor, Necroptosis, Tyrosine aminoacyl-tRNA synthetase (YARS)

## Abstract

**Background:**

Chemotherapy is the standard treatment for breast cancer; however, the response to chemotherapy is disappointingly low. Here, we investigated the alternative therapeutic efficacy of novel combination treatment with necroptosis-inducing small molecules to overcome chemotherapeutic resistance in tyrosine aminoacyl-tRNA synthetase (YARS)-positive breast cancer.

**Methods:**

Pre-chemotherapeutic needle biopsy of 143 invasive ductal carcinomas undergoing the same chemotherapeutic regimen was subjected to proteomic analysis. Four different machine learning algorithms were employed to determine signature protein combinations. Immunoreactive markers were selected using three common candidate proteins from the machine-learning algorithms and verified by immunohistochemistry using 123 cases of independent needle biopsy FFPE samples. The regulation of chemotherapeutic response and necroptotic cell death was assessed using lentiviral YARS overexpression and depletion 3D spheroid formation assay, viability assays, LDH release assay, flow cytometry analysis, and transmission electron microscopy. The ROS-induced metabolic dysregulation and phosphorylation of necrosome complex by YARS were assessed using oxygen consumption rate analysis, flow cytometry analysis, and 3D cell viability assay. The therapeutic roles of SMAC mimetics (LCL161) and a pan-BCL2 inhibitor (ABT-263) were determined by 3D cell viability assay and flow cytometry analysis. Additional biologic process and protein-protein interaction pathway analysis were performed using Gene Ontology annotation and Cytoscape databases.

**Results:**

YARS was selected as a potential biomarker by proteomics-based machine-learning algorithms and was exclusively associated with good response to chemotherapy by subsequent immunohistochemical validation. In 3D spheroid models of breast cancer cell lines, YARS overexpression significantly improved chemotherapy response via phosphorylation of the necrosome complex. YARS-induced necroptosis sequentially mediated mitochondrial dysfunction through the overproduction of ROS in breast cancer cell lines. Combination treatment with necroptosis-inducing small molecules, including a SMAC mimetic (LCL161) and a pan-BCL2 inhibitor (ABT-263), showed therapeutic efficacy in YARS-overexpressing breast cancer cells.

**Conclusions:**

Our results indicate that, before chemotherapy, an initial screening of YARS protein expression should be performed, and YARS-positive breast cancer patients might consider the combined treatment with LCL161 and ABT-263; this could be a novel stepwise clinical approach to apply new targeted therapy in breast cancer patients in the future.

## Background

Although approximately 70% of breast cancer patients are currently receiving standard chemotherapeutic regimens, the pathologic complete response (CR) rate is still low due to the high heterogeneity of breast cancers [1]. Therefore, a new strategy is required to overcome the low therapeutic efficacy of standard regimens and to avoid unnecessary complications caused by systemic chemotherapy.

Aminoacyl-tRNA synthetases (ARSs) have been described as being key to amino acid metabolism and found to induce necrosis [2, 3]. Generally, necrosis has been considered as an accidental cell death mechanism. However, recently accumulated evidence has indicated that necrosis is regulated by a programmed necrosis pathway called necroptosis [4]. The necroptotic pathway is induced by a series of receptor-interacting protein kinases (RIPK) and a mixed lineage kinase domain-like pseudokinase (MLKL) necrosome complex [5]. In contrast, the activity of the necrosome complex is inhibited by inhibitors of apoptosis proteins (IAP), leading to failure of necroptosis initiation. Therefore, the administration of IAP inhibitors, including second mitochondrial-derived activator of caspase (SMAC) mimetics (SM), to trigger necroptosis and overcome resistance to chemotherapy has been considered a promising strategy for treating several types of cancers [6]. Growing evidence also indicates that B cell lymphoma 2 (BCL2) functions as a robust anti-necroptotic protein [7], thereby conferring resistance to chemotherapy [8]. This has led to the recent development of selective BCL2 inhibitors, such as ABT-263 (navitoclax) and ABT-199 (venetoclax), for cancer treatment [9].

Here, we validated a novel anti-cancer therapeutic potency of the combined treatment with small molecules LCL161 and ABT-263 and relevant molecular mechanisms through the necrosome complex in tyrosine aminoacyl-tRNA synthetase (YARS)-positive breast cancer.

## Materials and methods

### Patient and clinical tissue sample selection

**Fig. 1:**
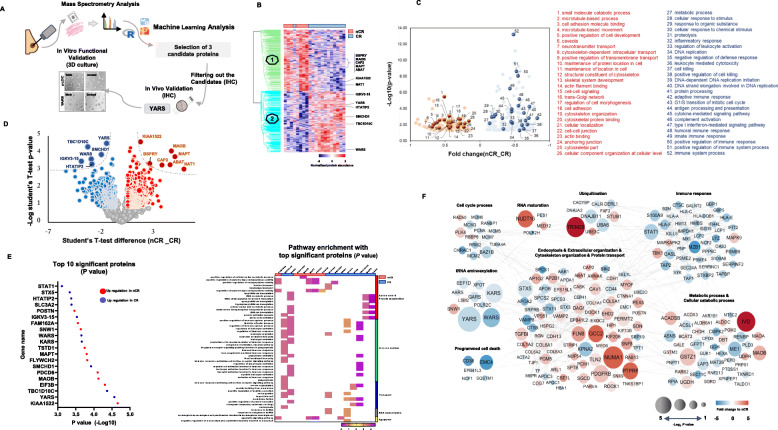
Aminoacyl-tRNA synthetases (ARSs) are vital proteins in breast cancer patients responsive to chemotherapy. **a** Overview of the study design. **b** Unsupervised hierarchical clustering analysis of proteins identified in the nCR and CR groups (top-ranked proteins were indicated as gene names). **c** Gene Ontology terms for biological process obtained using the ToppGene Suite gene list enrichment analysis. Red (#1 to #26) and blue (#27 to #52) indicate biological processes enriched in the nCR and CR group, respectively. **d** Differentially expressed proteins are depicted on volcano plots (the red dotted line indicates upregulation in nCR, and the blue dotted line indicates upregulation in CR: *P* < 0.05; the gray dotted line indicates FDR < 5%). **e** Pathway enrichment analysis of 20 top-ranked proteins showing statistical significance based on intensity ratio (*P* < 0.05, left: red dot, upregulation in nCR, and blue dot, upregulation in CR) and molecular function categories of each protein based on Gene Ontology biological process (right). **f** Overview of the protein interaction network model. Colors of nodes indicate the expression levels of quantified proteins. The thickness of the gray line indicates the protein-protein interaction score provided in the STRING public database (red circle, upregulation in nCR; blue circle, upregulation in CR). CR, complete remission; nCR, non-complete remission

Figure 1a indicates the key steps in our approach for the discovery of a novel biomarker and sensitizers. Pre-chemotherapeutic needle biopsy of 143 invasive ductal carcinomas with available post-chemotherapeutic surgical specimens for microscopic assessment of therapeutic effectiveness at the Seoul National University Hospital was enrolled under the approval of the Institutional Review Board at Seoul National University Hospital (IRB no. 1412-111-634). The baseline characteristics of the cases for proteomic analysis are summarized in Supplementary Table S1. Further details are available in Supplementary Materials (available online).

### LC-MS/MS proteomic analysis and peptide identification

Twenty FFPE samples were deparaffinized, and the peptide was digested using the filter-aided sample preparation (FASP) procedure as described previously [10]. Desalted pooled peptides were fractionated using the stage tip-based high-pH peptide fractionation method (PMID:24753479), then LC-MS/MS analysis was performed using a Q Exactive Plus Hybrid Quadrupole-Orbitrap mass spectrometer (Thermo Fisher Scientific Inc.), coupled to an Ultimate 3000 RSLC system (Dionex, Sunnyvale, CA, USA) via a nanoelectrospray source. Mass spectra were processed using a MaxQuant version 1.5.3.1 [11]. MS/MS spectra were searched by utilizing the Human UniProt protein sequence database (December 2014, 88,657 entries) using the Andromeda search engine with a 6-ppm precursor ion tolerance for total protein level analysis [12]. Experiment details are presented in the Supplementary Methods (available online).

### Label-free quantification and statistical analyses

For label-free quantification, the intensity-based absolute quantification (iBAQ) algorithm [13] was used as a part of the MaxQuant platform. Briefly, iBAQ values were calculated by MaxQuant as raw intensities divided by the number of theoretical peptides. Thus, iBAQ values are proportional to the molar quantities of the proteins. All statistical analyses were performed using the Perseus software [14]. Missing values were imputed based on a standard distribution (width = 0.15, downshift = 1.8) to simulate signals for proteins of low abundance. Finally, data were normalized using width adjustment, which subtracts medians and scales for all values in a sample to show equal interquartile ranges (Fig. 1c) [15]. Pairwise comparison of the proteomes included two-sided *t* tests performed utilizing threshold *P* value and a significance level of 5%. A protein was considered statistically significant if its fold change was ≥ 1.5 and *P* value ≤ 0.05.

### Machine learning analysis for predictive signatures

Determination of signature protein combinations utilized the concept of recursive feature elimination. Since recursive feature elimination selects a variable subset via machine learning model performance, we employed four different types of machine learning algorithms (naive Bayes classifier, random forest, SVM with polynomial kernel, and SVM with RBF kernel) from the *caret* package [16]. All algorithms have different hyper-parameters, and the training procedure for the *caret* package determines the optimum parameters by grid search. We performed leave-one-out cross-validation on the training set to classify samples between the CR and nCR groups, thus creating a list of potential signatures with the highest accuracy scores for each algorithm based on accuracy and AUC.

### Immunostaining

Immunoreactive markers were selected using three common candidate proteins from the machine learning algorithms subsequently validated by immunohistochemistry for 123 cases of independent needle biopsy FFPE samples which were obtained before chemotherapy. Standard immunohistochemistry procedures for the slides prepared by fixation in 10% neutral buffered formalin solution or 95% ethanol were performed using a benchmark automatic immunostaining device (Ventana BenchMark XT Staining System, Tucson, AZ, USA). The slides were incubated with anti-KIAA1522 (NBP1-90915, Novusbio) diluted 1:300, anti-PDCD6 (NBP1-19741, Novusbio) diluted 1:500, and anti-YARS (NBP1-86890, Novusbio) diluted 1:150. The immunohistochemical interpretation was evaluated by a semi-quantitative approach using an “*H*-score [17] in a blind and independent manner by two pathologists (H.J.O. and H.S.R.).

### Cell cultures and chemicals

T47D, MDA-MB-231, MDA-MB-468, and BT-20 cell lines were obtained from the American Type Culture Collection (ATCC; Manassas, VA, USA) and the Korea Cell Line Bank (KCLB, Korea). The T47D and BT20 cells were cultured in RPMI (Gibco, CA, USA) containing 10% fetal bovine serum (FBS; Invitrogen, Carlsbad, CA, USA) and 1% penicillin/streptomycin (PS; Gibco). MDA-MB-231 and MDA-MB-468 cells were cultured in DMEM (Gibco) containing 10% FBS and 1% PS. Cells were maintained at 37 °C in a humidified atmosphere of 95% air and 5% CO_2_ and periodically screened for *Mycoplasma* contamination. Both cells were confirmed by short tandem repeats (STR) DNA profiling tests in the Korean Cell Line Bank (KCLB). Caspase inhibitor z-VAD.fmk was purchased from R&D Systems, Inc. (Minneapolis, MN, USA), and SMAC mimetic LCL161 was purchased from Cayman Chemical (Ann Arbor, MI, USA). GSK’872 and necrosulfonamide (NSA) were purchased from Tocris Bioscience (Bristol, UK). ABT-263 (navitoclax) and ABT-199 (venetoclax) were obtained from Selleckchem (Houston, TX). Necrostatin-1 (Nec-1), docetaxel (DTX), Adriamycin (ADR), and cyclophosphamide (CPM) were purchased from Sigma-Aldrich (St. Louis, MO).

### Generation of lentiviral YARS overexpression cells

Lentiviral vectors encoding human YARS cDNA (Precision LentiORF, LOHS_100009313) and the control vector (encoding green fluorescent protein (GFP)) were used for YARS overexpression and purchased from Thermo Scientific (Loughborough, UK). Generation of the lentivirus and lentiviral vectors was co-transfected with pdPAX2 and pMD2.G (Addgene, MA, USA) into HEK293T cells (ATCC) using Lipofectamine 2000 (Lifetech, MA, USA). Supernatants were collected at 24 and 48 h and filtered in 0.45-μm pore syringes. T47D, MDA-MB-231, MDA-MB-468, and BT-20 cells were infected with the viral supernatant with 8 μg/ml polybrene, and stable cell lines were selected with blasticidin (range of 2~15 μg/ml).

### 3D cell viability assay

The effects of chemotherapeutic drugs on cell proliferation in 3-dimension (3D) were tested in a spheroid assay; 96-well plates were coated with Matrigel matrix (BD, Growth Factor Reduced), and cell suspensions were treated with 2% Matrigel and overlaid on precoated Matrigel. Cells were allowed to grow as spheroids for 2 days and then drug treated with various doses of drugs. Cells were preincubated with z-VAD.fmk for 1 h before treatment with SMAC mimetics (0.5–2 μM). For inhibitor assay, cells were preincubated with z-VAD.fmk (10 μM) or/and Nec-1 (50 μM), GSK’872 (10 μM), and NSA (1 μM) for 2 h before treatment with SMAC mimetics. For drug combination experiments, cells were treated with the indicated drugs, ABT-263 (1 μM or 0.5 μM), ABT-199 (1 μM), docetaxel (10 or 20 nM), Adriamycin (500 nM), cyclophosphamide (0.5 or 1 mM), and the SM/z-VAD.fmk treatment. 3D Cell viability was assessed by measuring the intracellular levels of ATP using the Cell Titer-Glo 3D luminescent cell viability assay kit (Promega). Luminescence was measured on a Luminometer (Glomax®Explore Multimode Microplate Reader, Promega, USA). Data were normalized to the control group (vector control or vehicle), and IC_50_ value calculations were made using Hill’s equation for GraphPad Prism software 8.

### LDH release assay

Cells were inoculated into 96-well tissue culture plates for 24 h; culture supernatants from each well were then transferred to new 96-well plates and mixed with 50 μL (1:1) of LDH solution (Thermo Fisher Scientific) at room temperature for 30 min in the dark. Absorbance was measured at 490 nm and 680 nm (ELISA Reader). The percentage of LDH released was then calculated.

### DEVDase activity assay

DEVDase activity in cells was determined using the Caspase-Glo 3/7 assay kit (Promega). Cells were seeded into 96-well opaque plate, media alone, or media containing drug (z-VAD.fmk) for 2 h. Caspase-Glo 3/7 reagent was added as 1:1 to each well and plates incubated at room temperature for 30 min before luminescence detection using a Luminometer (Glomax®Explore Multimode Microplate Reader, Promega, USA). Data were normalized to the control group (vector control or vehicle).

### Transmission electron microscopy (TEM)

Control and YARS cells were cultured and treated with H_2_O_2_ at the indicated concentration, harvested using 0.25% trypsin, and then washed with PBS. Then, the cells were collected by centrifugation for 10 min and treated as described by Huang et al. [18]. Briefly, the cells were fixed in ice-cold 2.5% glutaraldehyde in PBS (pH 7.3), rinsed with PBS, post-fixed in 1% osmium tetroxide with 0.1% potassium ferricyanide, dehydrated through a graded series of ethanol, and embedded in resin. The sections were stained with 1% uranyl acetate and 0.1% lead citrate and examined using a JEM2000EX transmission electron microscope (JEOL, Pleasanton, CA, USA).

### Oxygen consumption rate (OCR) analysis

The oxygen consumption rates of control and YARS cells were determined using the Seahorse XF Extracellular Flux Analyzer (Seahorse Bioscience Inc., North Billerica, MA). Briefly, cells were plated at a density of 40,000 cells/well (24-well plates (Seahorse Bioscience Inc)). The following day, the cells were washed, and fresh media were added. The sensor cartridge was loaded to dispense three metabolic inhibitors sequentially at specific time points: oligomycin (inhibitor of ATP synthase, 1 μM), followed by FCCP (a protonophore and uncoupler of mitochondrial oxidative phosphorylation, 0.5 μM), followed by the addition of a combination of R/A (mitochondrial complex I inhibitor, 1 μM). Basal oxygen consumption rate (OCR) was measured as well as the changes in oxygen consumption caused by the addition of the metabolic inhibitors described above. Several parameters were deducted from the changes in oxygen consumption, such as basal OCR and maximum mitochondrial capacity, as described previously [19].

### Small interfering RNA (siRNA) transfection and quantitative real-time PCR (qPCR)

RNA interference siRNAs targeting YARS and an AccuTarget Negative Control siRNA were purchased from Bioneer (Daejeon, Korea). Cells were transfected using Lipofectamine RNAiMAX (Invitrogen) following the manufacturer’s instruction. After incubation for 48 h, the YARS gene silencing was confirmed by assessing mRNA expression levels. Total RNA was isolated from the cell using the *AccuPrep*® Universal RNA Extraction Kit (Bioneer, Daejeon, Korea) with the manufacturer’s protocol. The genomic DNA was removed by DNase treatment using DNase-Free-DNase Set (Qiagen, Hilden, Germany). The cDNA was synthesized with *AccuPower*® RocketScript Cycle RT PreMix (Bioneer, Daejeon, Korea). Data analysis was based on the relative quantitative method, and ΔΔCT value was used to determine the relative fold change in the expression. All the data were normalized to the reference gene GAPDH expression level.

### Western blotting

Cells were collected and homogenized in RIPA lysis buffer (Thermo Fisher) on ice. Subsequently, the cell lysates were centrifuged at 4 °C to separate the proteins. Proteins were quantified using a Bicinchoninic Acid Protein Assay kit (Thermo Fisher). Western blotting analysis was performed using anti-RIPK, p-RIPK, RIPK3, p-RIPK3, MLKL (Cell signaling), and p-MLKL (Abcam) antibodies. Anti-GAPDH (BD Biosciences) antibodies were used as a loading control.

### Flow cytometry analysis

Cell apoptosis assay was performed using an Annexin V-FITC/propidium iodide (PI) apoptosis detection kit (BD Biosciences Pharmingen, San Diego, CA, USA). Briefly, cells were collected and washed twice with PBS and then suspended in 300 μl of binding buffer. Annexin V solution (5 μl) was added to the cell suspension and incubated for 15 min in the dark at room temperature. Subsequently, 200 μl of binding buffer and 5 μl of PI were added, and the cell suspension was immediately analyzed on BD FACSCaliber (BD Biosciences, San Jose, CA, USA). In the evaluation of mitochondrial reactive oxygen species (ROS), cells were trypsinized, pelleted, and incubated in mitoSOX® Red (Thermo Fisher Scientific) staining solution at 37 °C for 15 min and analyzed on a FACSCaliber. All data were processed with the FlowJo™ 10 software.

### Bioinformatics and statistical analysis

A statistical test of the proteome was performed using the Perseus software [14]. The iBAQ intensity values were transformed to log2 values, and a two-sample *t* test was performed with the significance of results considered at a *P* value of 0.05 and a 1.5-fold difference between two biological conditions. Gene Ontology annotation was performed using ToppGene Suite resources (https://toppgene.cchmc.org/) [20]. Interaction network models were constructed using Cytoscape ver3.7.0 [21]. All proteomic datasets were submitted to the ProteomeXchange Consortium (http://proteomecentral.proteomechange.org) via the PRIDE partner repository (accession number PXD013431) [22]. Annotated MS/MS spectra can be accessed through MS-Viewer [23]. Using the R package, we filtered candidate proteins for immunohistochemical validation using the Mann-Whitney *U* test to obtain variables showing significant differences between the two groups (*P* < 0.05). Statistical analysis of the results of in vitro tests was performed using the GraphPad Prism 8.0 program (GraphPad Software, Inc., CA, USA). Cell viability was measured using the CellTiter-Glo 3D luminescent assay kit, and *q* value calculations were made using Hill’s equation in the GraphPad Prism software 8.0. Student’s *t* test was used to determine the significance of the results (*P ≤ 0.05; **P ≤ 0.01; ***P ≤ 0.001).

## Results

### Aminoacyl-tRNA synthetases (ARS) as a key protein group in breast cancer patients responsive to chemotherapy

The unsupervised distribution of the samples filtered 476 from 6069 identified proteins in proteome analysis (Fig. 1b; Supplementary Fig. S1A; Supplementary Fig. S1B; Supplementary Table S2; Supplementary Table S3). The principal component analysis revealed the tight clustering of the two groups and distinct protein expression patterns within each group (Supplementary Fig. 1C). Analysis of pathway enrichment using the Gene Ontology database showed that several commonly enriched pathways, including those for the cell adhesion process, cytoskeletal organization process, vesicle organization process, and Golgi organization process, were overrepresented in a group of samples from patients with breast cancer showing poor response to the chemotherapy (Fig. 1c). On the contrary, multiple immune response-related processes and aminoacylation processes were primarily represented in a group of samples from patients who showed complete remission (CR, Fig. 1c).

By applying a more stringent statistic cutoff (FDR < 5%) to obtain reliable protein candidates, ARSs, including YARS and tryptophanyl-tRNA synthetase (WARS), were consistently selected as key proteins in breast cancer patients showing good response to chemotherapy (Fig. 1d). Gene Ontology analyses using top-ranked proteins based on *P* value enriched 20 significantly altered gene sets, including ARSs, and distinct biological functions, such as peptide metabolic processes and protein translation (Fig. 1e). Protein-protein interaction revealed a tight cluster of tRNA aminoacylation-related proteins that are completely downregulated in breast cancer patients with poor response to chemotherapy (Fig. 1f).

### YARS protein expression predicts chemotherapeutic response in breast cancer patients

**Fig. 2:**
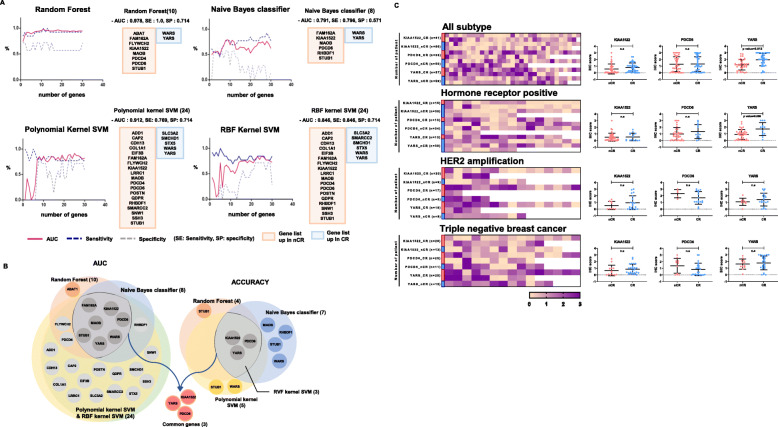
YARS protein expression is validated as a predictor of chemotherapeutic response in breast cancer. **a** Comparison of the predictive powers of the combinations of the best candidate proteins determined using machine learning algorithms for predicting chemotherapy response. The predictive performance of each algorithm to predict chemotherapy response is represented by a linear plot along with AUC, sensitivity, and specificity (red square, upregulated proteins in the nCR group; blue square, upregulated proteins in the CR group). **b** A convergent filtering approach utilizing predictive values to select the candidate with potential therapeutic value. **c** Needle biopsy FFPE tissues from a set of 123 independently enrolled patients were used to verify the predictive performance of KIAA1522 or YARS; 79 patients were enrolled to confirm the predictive performance of PDCD6 for all intrinsic subtypes of breast cancer. Subset analysis based on intrinsic subtypes, including luminal, HER2-enriched, and triple-negative breast cancer (left). The heatmap demonstrates negative to gradient staining, representing the *H*-score values for proteins. The graph provided next to the heatmap demonstrates an intensity ratio of immunohistochemical staining between the nCR and CR groups (right, Mann-Whitney *U* test). CR, complete remission; nCR, non-complete remission

To select proteins predicting chemotherapeutic responses in breast cancer, we performed machine learning analysis using four types of algorithms using 4170 quantified proteins from proteomic data (Supplementary Fig. S1B; Supplementary Table S4). The machine learning approach employing the random forest algorithm demonstrated the highest AUC value, 0.978 (sensitivity 1.0 and specificity 0.714) with a combination of 10 proteins, including STUB1, PDCD6, MAOB, PDCD4, PDCD6, FLYWCH2, ABAT, FAM162A, YARS, and WARS (Fig. 2a; Supplementary Table S5). Feature selection based on AUC value demonstrated seven common proteins, including STUB1, PDCD6, YARS, MAOB, FAM162A, KIAA 1522, and WARS, whereas three proteins, including PDCD6, YARS, and KIAA 1522, were selected from an additional prediction model for the accuracy value (Fig. 2b). Comparison of results obtained using the two approaches suggested three common proteins, including one favorable target, YARS, and two unfavorable targets, KIAA1522 and PDCD6, as the most reliable proteins to predict chemotherapeutic responses in breast cancer (Fig. 2b).

These three prioritized proteins were further evaluated in an independent validation set of 123 patients using immunohistochemistry. Nonparametric analysis using the Mann-Whitney *U* test indicated an association between the higher abundance of YARS and complete remission in response to chemotherapy (*P* = 0.012, Fig. 2c; Supplementary Fig. S4).

### YARS sensitizes breast cancer to standard chemotherapeutic agents by inducing the necrotic cell death pathway

**Fig. 3:**
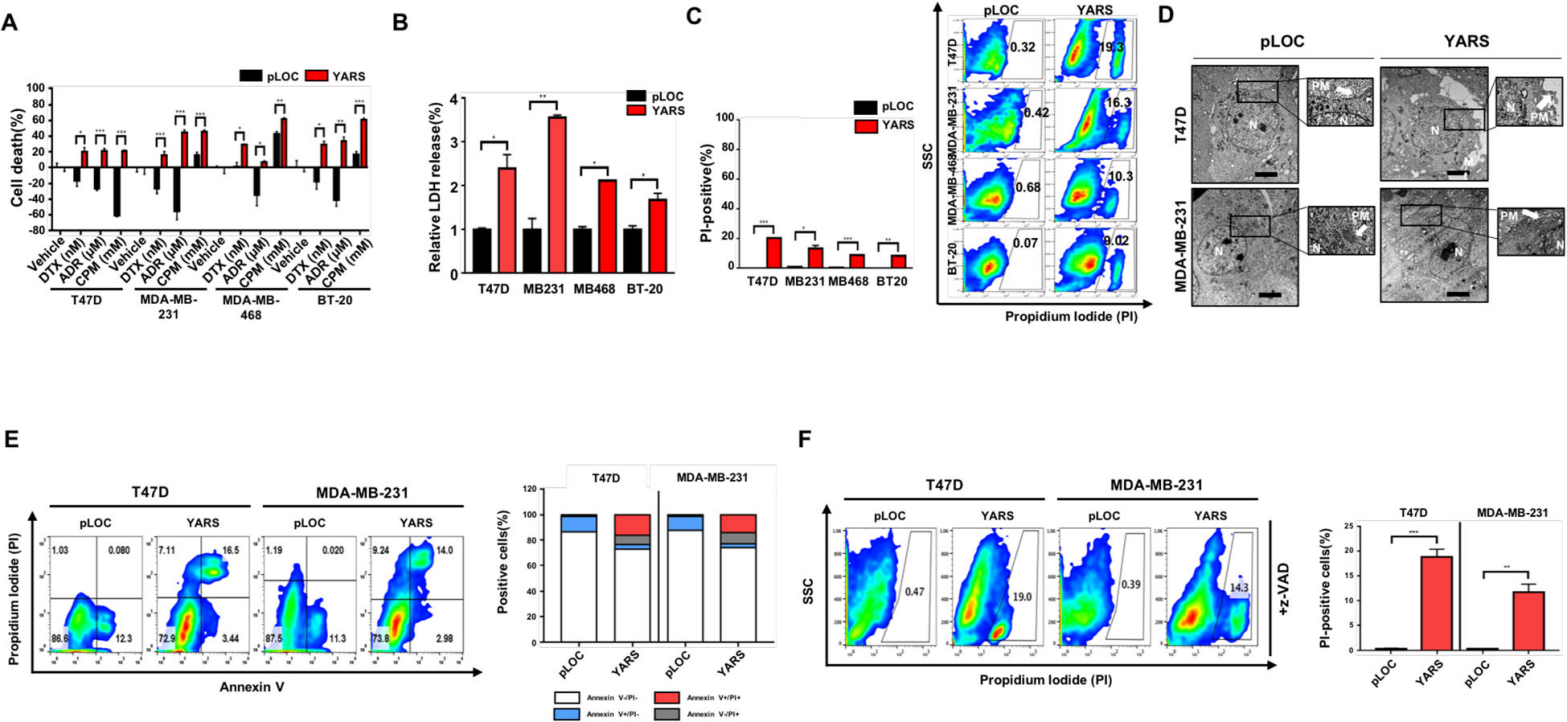
YARS sensitizes breast cancer cells to standard chemotherapeutic agents by inducing the necrotic cell death pathway. **a** The cytotoxicity effect of docetaxel (DTX), Adriamycin (ADR), and cyclophosphamide (CPM) on YARS-overexpressing 3D spheroid models. **b** Cell death was determined using the LDH release assay for YARS-overexpressing and control cells. **c** Representative graphs of plasma membrane integrity determined via propidium iodide (PI) staining followed by flow cytometry. **d** Representative images of YARS-induced morphological changes captured using a transmission electron microscope. The scale bar represents 5 μm. **e** Cell death was determined using Annexin V/PI-staining and flow cytometry (left). Representative graphs show the Annexin-V- and/or PI-positive and PI-negative distributions of cells (right). **f** Plasma membrane integrity was determined through PI staining followed by flow cytometry (left). Representative graphs for cell death determined using PI staining (right). All cells were pretreated with pan-caspase inhibitor z-VAD.fmk (10 μM) for 1 h and then examined for PI-positive cells

Since YARS overexpression was verified as a strong biomarker predicting good response to chemotherapy in the breast cancer patient cohort, we established YARS-overexpressing cell models (Supplementary Fig. S2) to explore further the biological functions of YARS and how it affects chemotherapeutic efficacy. The 3D spheroid cytotoxicity assay showed that YARS upregulation dramatically reduced cell viability in breast cancer cell lines receiving chemotherapy (*P* value from 0.036 to < 0.001, Fig. 3a; Supplementary Fig. S5).

We next explored the cell death mechanism induced by YARS in breast cancer cells*.* The lactate dehydrogenase (LDH) release assay showed that YARS significantly promoted LDH release up to 3.5-fold (Fig. 3b), and FACS analysis demonstrated 8- to 20-fold increases in PI-positive populations, which consistently indicated that YARS damaged the plasma membrane in breast cancer cells (Fig. 3c). Transmission electron microscopy (TEM) also revealed that YARS upregulation caused structural alterations, including the loss of normal plasma membrane integrity, which is a morphologic characteristic of necrosis (Fig. 3d). Flow cytometric analysis also showed that YARS increased the proportion of Annexin V−/PI+ and Annexin V+/PI+ cells, indicating late apoptosis or necrosis (Fig. 3e). To distinguish cells undergoing late apoptosis and necrosis, additional PI staining of breast cancer cells was performed after treatment with the pan-caspase apoptotic inhibitor z-VAD.fmk [24]. Consequently, YARS was found to have significantly increased the proportion of PI+ necrotic cells in the presence of z-VAD.fmk (Fig. 3f; Supplementary Fig. S6). Together, these findings indicated that YARS induced cancer cell death via caspase-independent necrosis in breast cancer.

### YARS-induced necroptotic cell death is accompanied by reactive oxygen species (ROS) production and mitochondrial dysfunction in breast cancer

**Fig. 4:**
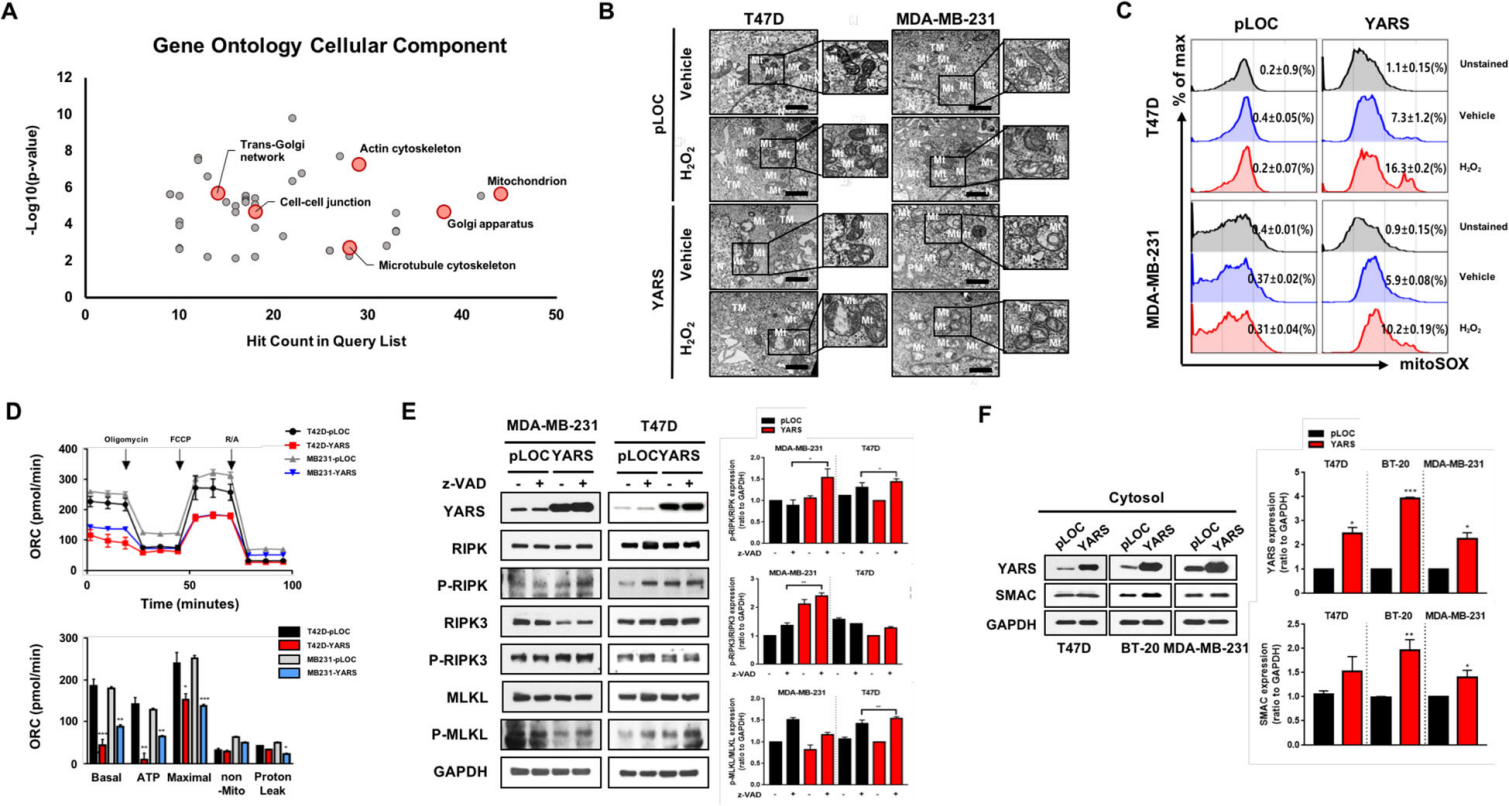
YARS induces necroptotic cell death through reactive oxygen species (ROS) production and mitochondrial dysfunction in breast cancer. **a** The subcellular components enriched in the Gene Ontology analysis between CR and nCR. **b** The mitochondrial morphologies of control (pLOC) and YARS-overexpressing cells captured using transmission electron microscopy (TEM) in the presence or absence of H_2_O_2_. The scale bar represents 1 μm. **c** Representative graphs of mitoSOX flow cytometry analysis in the presence or absence of H_2_O_2_. **d** Real-time measurements of the mitochondrial oxygen consumption rates (OCRs) indicating the mitochondrial biogenetic profile (top). Quantification of the basal OCR, ATP-linked OCR, maximal OCR, nonmitochondrial OCR, and proton leak (bottom). **e** Protein levels of RIPK, p-RIPK, RIPK3, p-RIPK3, MLKL, and p-MLKL were assessed in the presence or absence of z-VAD.fmk in MDA-MB-231 and T47D cells by western blotting (left), and western blot bands were quantified by densitometry using the ImageJ Lab software (right). **f** Comparative western blot analyses of YARS and SMAC expression in the cytosolic fractions of control and YARS cells (T47D, BT-20, and MDA-MB-231, left) and western blot band densities (right). Three independent western blots were quantified by densitometry using the ImageJ Lab software. GAPDH served as a loading control. Data are expressed as the mean ± SEM of triplicate experiments; *P < 0.05; **P < 0.01; ***P < 0.001

We next explored how YARS induced necrosis in breast cancer cells. Cellular components Gene Ontology analysis of chemotherapeutic response (CR vs. nCR) from a breast cancer patient cohort annotated 44 proteins in the mitochondria, which was the highest number compared to all remaining subcellular organelles (*n* = 44, Fig. 4a). TEM also showed structural disruption of mitochondria in YARS-overexpressing breast cancer cells, such that the mitochondria had disorganized membranes and loss of cristae (Fig. 4b).

Since damaged mitochondria upregulate reactive oxygen species (ROS) production, leading to oxidative stress and suppression of ATP generation through OXPHOS in mitochondria [25], we assumed that YARS may disrupt the mitochondrial structure and reduce energy production via loss of ROS detoxification. Flow cytometry analysis using mitoSOX red staining showed 7.3- and 5.9-fold increases in the production of mitochondrial superoxide in YARS-induced T47D and MDA-MB-231 cells, respectively, compared to YARS-negative breast cancer cells (Fig. 4c). YARS-positive breast cancer cells generated a 2-fold higher level of mitochondrial superoxide in the presence of H_2_O_2_ (Fig. 4c). The average oxygen consumption rate (OCR) was significantly decreased in YARS-overexpressing breast cancer cells (*P* < 0.01, Fig. 4d). The values of ATP-linked OCR, maximal OCR, and reserve capacity were all dramatically reduced in YARS-positive breast cancer cells compared to YARS-negative breast cancer cells (*P* < 0.01, Fig. 4d). Altogether, these data indicated that YARS overexpression mediates mitochondrial ROS accumulation, leading to a remarkable reduction of ATP production in breast cancer.

Based on the results described above and growing evidence from previous studies concerning the central role of mitochondrial ROS leading to necroptosis [26], we next explored whether changes in the expression of YARS induces necroptosis in breast cancer cells. The key necroptosis molecules, including RIPK and RIPK3, were phosphorylated in YARS-positive MDA-MB-231 cells upon z-VAD.fmk (Fig. 4e). And RIPK and MLKL were phosphorylated in YARS-overexpressed T47D cells treated with z-VAD.fmk (Fig. 4e). Since SMAC is a protein released from the mitochondria into the cytoplasm during necroptosis [27], we performed an additional blotting assay using cytosol fractionation to identify the cytosolic level of SMAC in YARS-induced breast cancer cells. The level of cytosolic SMAC was increased in the YARS-positive group compared to that in pLOC cells (Fig. 4f). Collectively, these data indicated that YARS mediates the excessive release of SMAC from the mitochondria into the cytoplasm, leading to breast cancer cell death by triggering necroptosis.

### Combination treatment with SM/z-VAD and BCL2 inhibitor suppressed tumor growth and viability via phosphorylation of necrosome complex in YARS-positive breast cancer cells

**Fig. 5:**
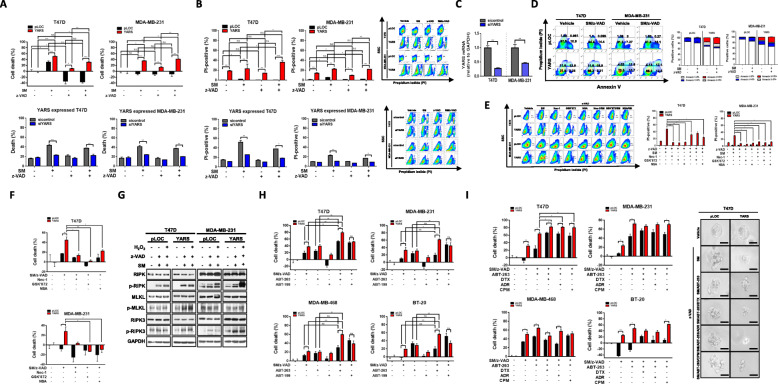
Combination treatment with SM/z-VAD and BCL2 inhibitor promotes tumor death via phosphorylation of necrosome complex in YARS-positive breast cancer cells. **a** The cytotoxicity effect of SM (T47D, 0.5 μM; MDA-MB-231, 2 μM) in the absence or presence of 10 μM z-VAD.fmk on 3D spheroid models. Cell death was assessed using 3D CellTiter-Glo Luminescent Cell Viability (top). YARS knockdown cells were treated with SM for 24 h in the absence or presence of 10 μM z-VAD.fmk, and cell death was determined by FSC/SSC analysis and flow cytometry (bottom). **b** Cells were treated with SM for 24 h in the absence or presence of z-VAD.fmk. Cell death was determined by PI staining followed by flow cytometry (right). Representative graphs of cell death determined using PI staining (left). YARS knockdown cells were treated with SM for 24 h in the absence or presence of 10 μM z-VAD.fmk., and cell death was determined by PI staining followed by flow cytometry (lower right). Representative graphs of cell death determined using PI staining (left). **c** The mode of cell death after combination treatment with SM/z-VAD.fmk was determined by Annexin V/PI staining and flow cytometry (left). Representative graphs show the distribution of Annexin-V- and/or PI-positive and PI-negative cells (right). **d** Cells were pre-stimulated with DMSO (control), z-VAD.fmk (10 μM) and/or Nec-1 (50 μM), GSK’872 (5 μM), and NSA (1 μM) in several combinations for 2 h followed by stimulation with SM for a further 24 h. Cell death was determined by PI staining and flow cytometry (left). The representative graph shows the distribution of PI-positive cells for each drug combination (right). In **a**–**d**, the mean ± SD of at least three independent experiments performed in triplicate are shown; *P < 0.05; **P < 0.01; ***P < 0.001. **e** 3D spheroids were treated for 24 h with SM (T47D, 0.5 μM; MDA-MB-231, 2 μM) in the presence of 10 μM z-VAD.fmk and/or 50 μM Nec-1, 5 μM GSK’872, or 1 μM NSA. Cell death was assessed by 3D CellTiter-Glo Luminescent Cell Viability. **f** The protein levels of RIPK, p-RIPK, RIPK3, p-RIPK3, MLKL, and p-MLKL were assessed by western blotting in the presence of SM/z-VAD and/or H_2_O_2_. GAPDH served as a loading control. **g** Drug combination experiments assessing the effect of the treatment of 3D spheroids with the indicated drugs, ABT-263 (T47D, 1 μM; MDA-MB-231, 0.5 μM) and ABT-199 (1 μM), combined with SM/z-VAD.fmk treatment on YARS-induced cell death. Cell death was assessed using 3D CellTiter-Glo Luminescent Cell Viability. **h** For determining the combined effect of ABT-263 and SM/z-VAD.fmk on the YARS-induced necroptosis 3D spheroid models, the models were treated with ABT263 (T47D, 1 μM; MDA-MB-231, 0.5 μM) and/or DTX (T47D, 5 nM; MDA-MB-231, 10 nM), ADR (500 nM), or CPM (T47D, 0.5 mM; MDA-MB-231, 1 mM). 3D cell viability was analyzed using a CellTiter-Glo Luminescent Cell Viability Assay Kit (left), and T47D-derived spheroid images were assessed using phase-contrast microscopy (right). Data (**e**, **g**, **h**) shown are the mean ± SEM with *n* = 4; *P < 0.05; **P < 0.01; ***P < 0.001

Since we identified the release of a mitochondrial necrosis inducer SMAC in YARS-overexpressed breast cancer cells and growing evidence has shown SM as a novel targeted anti-cancer drug [28], 3D spheroid necroptosis models were used to evaluate the therapeutic efficacy of SM in breast cancer. SM/z-VAD.fmk treatment reduced the cell viability of YARS-overexpressing breast cancer cells (Fig. 5a). On the contrary, the survival of YARS-knockdown cell lines was restored regardless of SM/z-VAD.fmk treatment (Fig. 5a). FACS analysis demonstrated concordant findings of increased cell death (PI-positive) in YARS-overexpressing cells and decreased cell death in YARS-knockdown cells after SM/z-VAD.fmk treatment (Fig. 5b). YARS knockdown was confirmed by qPCR (Fig. 5c). Further flow cytometry analysis showed decreased Annexin V+/PI− early apoptotic populations and increased Annexin V+/PI+ necroptotic populations after SM/z-VAD.fmk treatment (Fig. 5d). Next, we treated YARS-overexpressing breast cancer cells with necroptosis inhibitors, including Nec-1 (inhibitor of RIPK), GSK’872 (inhibitor of RIPK3), and NSA (inhibitor of MLKL kinase), after SM/z-VAD.fmk treatment to confirm whether SM promotes necroptosis compared to apoptosis. Necroptosis inhibitors that selectively inactivate necrosome complex kinase proteins consistently reduced the PI-positive cell death population in YARS-overexpressing cell lines (Fig. 5e). Moreover, all necroptosis inhibitors restored cell survival in YARS-overexpressing 3D spheroid models treated with SM/z-VAD.fmk (Fig. 5f). The activation of several necrosome complex kinase proteins was also confirmed by the increase in their phosphorylation in cell lines upon SM/z-VAD.fmk treatment (Fig. 5g).

Recently, studies demonstrated that BCL2 plays a role as an anti-necroptotic protein [7]. In The Cancer Genome Atlas (TCGA, *n* = 996) and METABRIC datasets (*n* = 2173), *BCL2* mRNA expression showed a significant negative correlation with MLKL expression (*ρ* = − 0.39, *P* ≤ 0.001, TCGA) and marginal negative correlation with RIPK expression (*ρ* = − 0.0352, *P* = 0.125, METABRIC, Supplementary Fig. S3). Therefore, we assumed that therapeutic efficacy could be expected with combined treatment using a BCL2 inhibitor, such as ABT-263 (Navitoclax) and ABT-199 (Venetoclax), and an SM (LCL161) in YARS-positive breast cancer. Combined treatment with SM/z-VAD.fmk and ABT-263 showed that it had the highest efficacy as a regimen for the YARS-positive 3D spheroid model (Fig. 5h). ABT-199 has no synergistic effect with SM/z-VAD.fmk (Fig. 5h). Moreover, among the three major proteins of the necrosome complex, p-RIPK protein expression was specifically increased by treatment with SM/z-VAD and ABT-263 in YARS-positive breast cancer cells; the expression of this protein increased further upon additional treatment with H_2_O_2_ in the MDA-MB-231 cell line (Supplementary Fig. S6). Together, these findings indicated that the therapeutic potency of the combination of the small molecules, SM/z-VAD and ABT-263, in YARS-overexpressing breast cancer is achieved through a necroptosis signaling pathway.

We further verified the synergistic effects of SM/z-VAD and ABT-263 in the standard chemotherapeutic regimen. Combined treatment with small molecules and chemotherapy reduced cell viability in the YARS-positive 3D tumor spheroid model (Fig. 5i). The synergic effect of standard chemotherapeutic agents in combination with SM/v-VAD and ABT-263 was the most prominent in the hormone receptor-positive T47D cell line (*P* = from 0.004 to 0.048, Fig. 5h). A partial cytotoxic effect was also observed in the spheroid models of hormone receptor-negative cell lines, depending on the combination of therapeutic agents used (Fig. 5i). Along with the synergic effect of the small molecules on chemotherapy, treatment with SM/v-VAD and ABT-263 without chemotherapeutic agents showed stronger cytotoxic effects compared to those observed in response to combined treatment with SM/v-VAD and ABT-263 with chemotherapeutic agents in YARS-overexpressing breast cancer cell lines (*P* = from 0.003 to 0.029, Fig. 5i). Collectively, our results indicated that SM/z-VAD and ABT-263 have therapeutic potential as novel targeted drugs that are comparable with chemotherapeutic efficacy and a partial synergistic effect on the standard chemotherapeutic regimen in YARS-positive breast cancer.

## Discussion

**Fig. 6:**
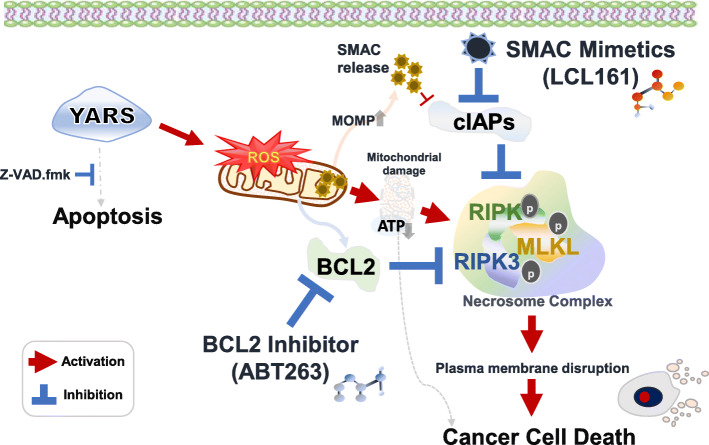
The proposed mechanism underlying the synergistic effects of LCL161 (SMAC mimetic) and ABT-263 (Navitoclax, pan-BCL2 inhibitor) through YARS-induced necroptosis in breast cancer. Upon overexpression of YARS, the accumulation of ROS in the mitochondria, inducing mitochondrial outer membrane permeabilization (MOMP), is triggered; this is followed by the release of SMAC into the cytosol and reduced ATP generation. LCL161 (SMAC mimetic), a cIAPs antagonist, induces cell death through caspase activation. The LCL161-mediated depletion of cIAPs proteins leads to necroptosis. During necroptosis, RIPK interacts with RIPK3 and MLKL to form the necrosome complex, which promotes the activation of RIPK3 and MLKL; subsequently, MLKL oligomerizes and translocates to and forms pores in the plasma membrane, leading to plasma membrane disruption. ABT-263 directly binds to and neutralizes pro-survival BCL2-like proteins, aiding the induction of necroptosis. The synergistic effect of LCL161 and ABT-263 leads to the phosphorylation of the necrosome complex, and inhibition of pan-caspase might have implications as a new therapeutic modality for treatment in breast cancer

Here, we demonstrate that combination treatment, including SM/z-VAD and the BCL2 inhibitor ABT-263 (Navitoclax), results in cancer cytotoxic effects and synergistic effect on the standard chemotherapeutic regimen by phosphorylation of the necrosome complex in YARS-positive breast cancer (Fig. 6). Our immunohistochemical verification cohort of breast cancer patients selected based on a preselection of the most relevant feature via machine learning algorithms, consistently demonstrated YARS as the most reliable marker for predicting the response to chemotherapy. Mechanistically, in a 3D spheroid model that mimics the in vivo environment, the overexpression of YARS dramatically increased the chemotherapeutic effect in breast cancer cell lines. YARS initially increased mitochondrial free ROS. Although the functional relevance of the increase in ROS by YARS is not fully understood, previous studies have shown the regulatory effect of the ARS complex on ROS-mediated cell death [29–31]. In our study, mitoSOX flow cytometry and electron microscopic analysis further demonstrated that the YARS-induced ROS production mainly occurs in the mitochondria. Growing evidence has also shown that mitochondrial oxygen species sequentially contribute to the process of necroptosis through the phosphorylation of the necrosome complex [26], which is concordant with our findings. Our data also showed that ROS activated major necrosome complex cascade genes, such as RIPK, RIPK3, and MLKL, especially in YARS-overexpressing breast cancer cell lines. We observed more specific phosphorylation of RIPK after the treatment of these cell lines with SM and ABT-263 than remaining kinase proteins. Under resting conditions, RIPK binds to RIPK3 and inactivates RIPK3-mediated necroptosis [32]. However, upon death signaling stimulation, the functional interplay between RIPK and RIPK3 recruits MLKL to trigger downstream necroptosis. Thus, RIPK is suggested to be a crucial kinase protein for the initial formation of the necrosome complex [32]. Therefore, we assume that RIPK might play a pivotal role by being initially targeted in the combined treatment with SM and ABT-263 in YARS-positive breast cancer. Along with the activation of the necrosome complex by targeting BCL2 and SMAC, downregulation of BCL2 increases the permeability of the outer mitochondrial membrane, allowing cytochrome c and SMAC to be released from the mitochondria into the cytosol, and caspase-independent cell death [33]. Therefore, LCL161 and ABT-263 are newly developed necroptosis inducers, and multiple clinical trials are underway to overcome drug resistance by antagonizing IAP proteins and BCL2 protein that is known to be linked to therapeutic failure and unfavorable prognosis in malignant tumors [7, 8, 34]. A recent preclinical model of lymphoma exhibited synergistic antitumor activity of LCL161 with rituximab in both in vivo and in vitro experimental models of various types of lymphomas with the chemotherapy resistance [35]. A randomized clinical trial for the use of LCL161 and everolimus to treat malignant tumors, including triple-negative breast cancer, is currently underway (clinical trial identifier: NCT02890069).

ABT-263, also known as navitoclax, is an orally bioavailable anti-cancer drug that showed a therapeutic effect in breast cancer [36]. A phase II clinical trial further demonstrated a potent therapeutic activity of ABT-263 against advanced lung cancer [37]. Our study first showed the synergistic effects of a BCL2 inhibitor ABT-263 and SM/z-VAD.fmk LCL161 with the aim of identifying a reinforcing anti-cancer effect that can be utilized as novel targeted agents in YARS-positive breast cancer.

## Conclusions

The proteome-level data obtained by us provide a stepwise clinical procedure in YARS-positive breast cancer patients, which involves an initial screening test for YARS, followed by treatment of YARS-positive breast cancer patients with SM and BCL2 inhibitors. We confirmed comparable therapeutic efficacies of the combination of small molecules and conventional chemotherapy and suggested a new therapeutic modality for breast cancer patients showing resistance to conventional chemotherapy. While further in vivo validation study is required, we anticipate that this novel combined therapy may be clinically applicable to breast cancer patients in the future.

## Supplementary Information

**Additional file 1.** “BCR_revision Figure.

**Additional file 2.** Supplementary table.

**Additional file 3.** Supplementary File.

## Data Availability

The mass spectrometry proteomic data have been deposited to the ProteomeXchange Consortium (http://proteomecentral.proteomexchange.org) via the PRIDE [22] partner repository with the dataset identifier PXD013431 (https://www.ebi.ac.uk/pride/archive/projects/PXD013431). Annotated MS/MS spectra can be accessed through MS-Viewer (http://msviewer.ucsf.edu/prospector/cgi-bin/mssearch.cgi?report_title=MS-Viewer&search_key=d4qfuhxipu& search_name=msviewer) with the following search keys: IKE0jhYd. Project name: In-depth proteomic analysis for discovery of response prediction markers in breast cancer chemotherapy using needle biopsy FFPE tissue. Project ID: PXD013431.

## Abbreviations

ARSs: Aminoacyl-tRNA synthetases
AUC: Area under the ROC curve
BCL2: B cell lymphoma 2
CR: Complete response
FDR: False discovery rate
IAP: Inhibitors of apoptosis proteins
LDH: Lactate dehydrogenase
MLKL: Mixed lineage kinase domain-like pseudokinase
OCR: Oxygen consumption rate
RIPK: Receptor-interacting protein kinases
pLOC: Precision LentiORF
ROS: Reactive oxygen species
SM: SMAC mimetic
SMAC: Second mitochondrial-derived activator of caspase
TEM: Transmission electron microscopy
WARS: Tryptophanyl-tRNA synthetase
YARS: Tyrosine aminoacyl-tRNA synthetase
