# Supplementary material for: Combined the SMAC mimetic and BCL2 inhibitor sensitizes neoadjuvant chemotherapy by targeting necrosome complexes in tyrosine aminoacyl-tRNA synthase-positive breast cancer
Source: Breast Cancer Res. 2020 Nov 25;22:130. doi: 10.1186/s13058-020-01367-7 (PMC7687715; doi:10.1186/s13058-020-01367-7)

Figure 1

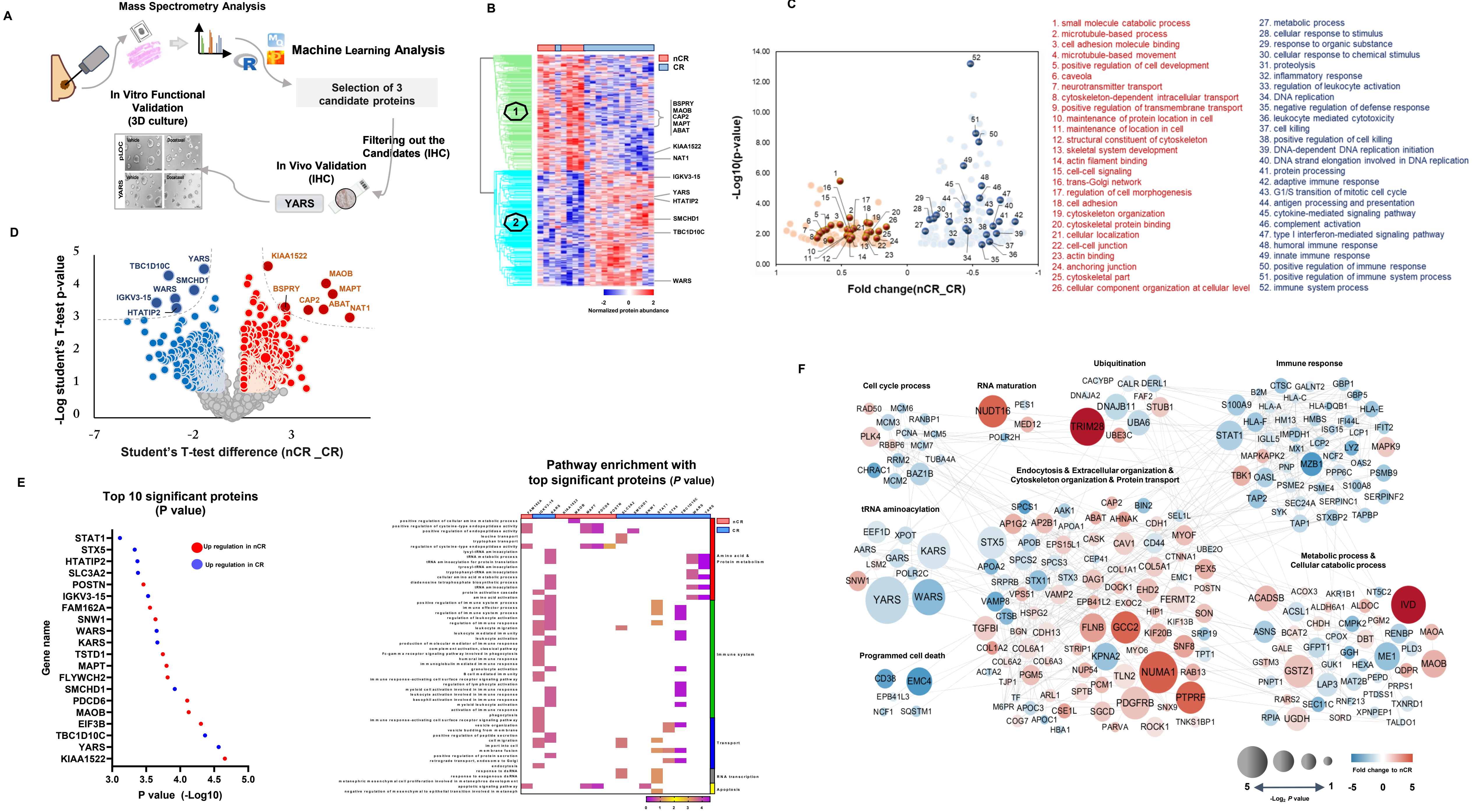
A
Mass Spectrometry Analysis
Machine Learning Analysis
Selection of 3 candidate proteins
Filtering out the Candidates (IHC)
In Vivo Validation (IHC)
YARS
In Vitro Functional Validation (3D culture)
pLOC
YARS
Vehicle
Docetaxel
B
nCR
CR
1
2
BSPRY
MAOB
CAP2
MAPT
ABAT
KIAA1522
NAT1
IGKV3-15
YARS
HTATIP2
SMCHD1
TBC1D10C
WARS
Normalized protein abundance
C
-Log10(p-value)
Fold change(nCR_CR)
1. small molecule catabolic process
2. microtubule-based process
3. cell adhesion molecule binding
4. microtubule-based movement
5. positive regulation of cell development
6. caveola
7. neurotransmitter transport
8. cytoskeleton-dependent intracellular transport
9. positive regulation of transmembrane transport
10. maintenance of protein location in cell
11. maintenance of location in cell
12. structural constituent of cytoskeleton
13. skeletal system development
14. actin filament binding
15. cell-cell signaling
16. trans-Golgi network
17. regulation of cell morphogenesis
18. cell adhesion
19. cytoskeleton organization
20. cytoskeletal protein binding
21. cellular localization
22. cell-cell junction
23. actin binding
24. anchoring junction
25. cytoskeletal part
26. cellular component organization at cellular level
27. metabolic process
28. cellular response to stimulus
29. response to organic substance
30. cellular response to chemical stimulus
31. proteolysis
32. inflammatory response
33. regulation of leukocyte activation
34. DNA replication
35. negative regulation of defense response
36. leukocyte mediated cytotoxicity
37. cell killing
38. positive regulation of cell killing
39. DNA-dependent DNA replication initiation
40. DNA strand elongation involved in DNA replication
41. protein processing
42. adaptive immune response
43. G1/S transition of mitotic cell cycle
44. antigen processing and presentation
45. cytokine-mediated signaling pathway
46. complement activation
47. type I interferon-mediated signaling pathway
48. humoral immune response
49. innate immune response
50. positive regulation of immune response
51. positive regulation of immune system process
52. immune system process
D
-Log student's T-test p-value
Student's T-test difference (nCR _CR)
TBC1D10C
YARS
SMCHD1
WARS
IGKV3-15
HTATIP2
KIAA1522
BSPRY
MAOB
MAPT
CAP2
ABAT
NAT1
E
Top 10 significant proteins (P value)
Up regulation in nCR
Up regulation in CR
Gene name
P value (-Log10)
STAT1
STX5
HTATIP2
SLC3A2
POSTN
IGKV3-15
FAM162A
SNW1
WARS
KARS
TSTD1
MAPT
FLYWCH2
SMCHD1
PDCD6
MAOB
EIF3B
TBC1D10C
YARS
KIAA1522
Pathway enrichment with top significant proteins (P value)
Amino acid & Protein metabolism
Immune system
Transport
RNA transcription
Apoptosis
F
Cell cycle process
RNA maturation
Ubiquitination
Immune response
tRNA aminoacylation
Endocytosis & Extracellular organization & Cytoskeleton organization & Protein transport
Metabolic process & Cellular catabolic process
Programmed cell death
Fold change to nCR
-Log2 P value

Figure 2

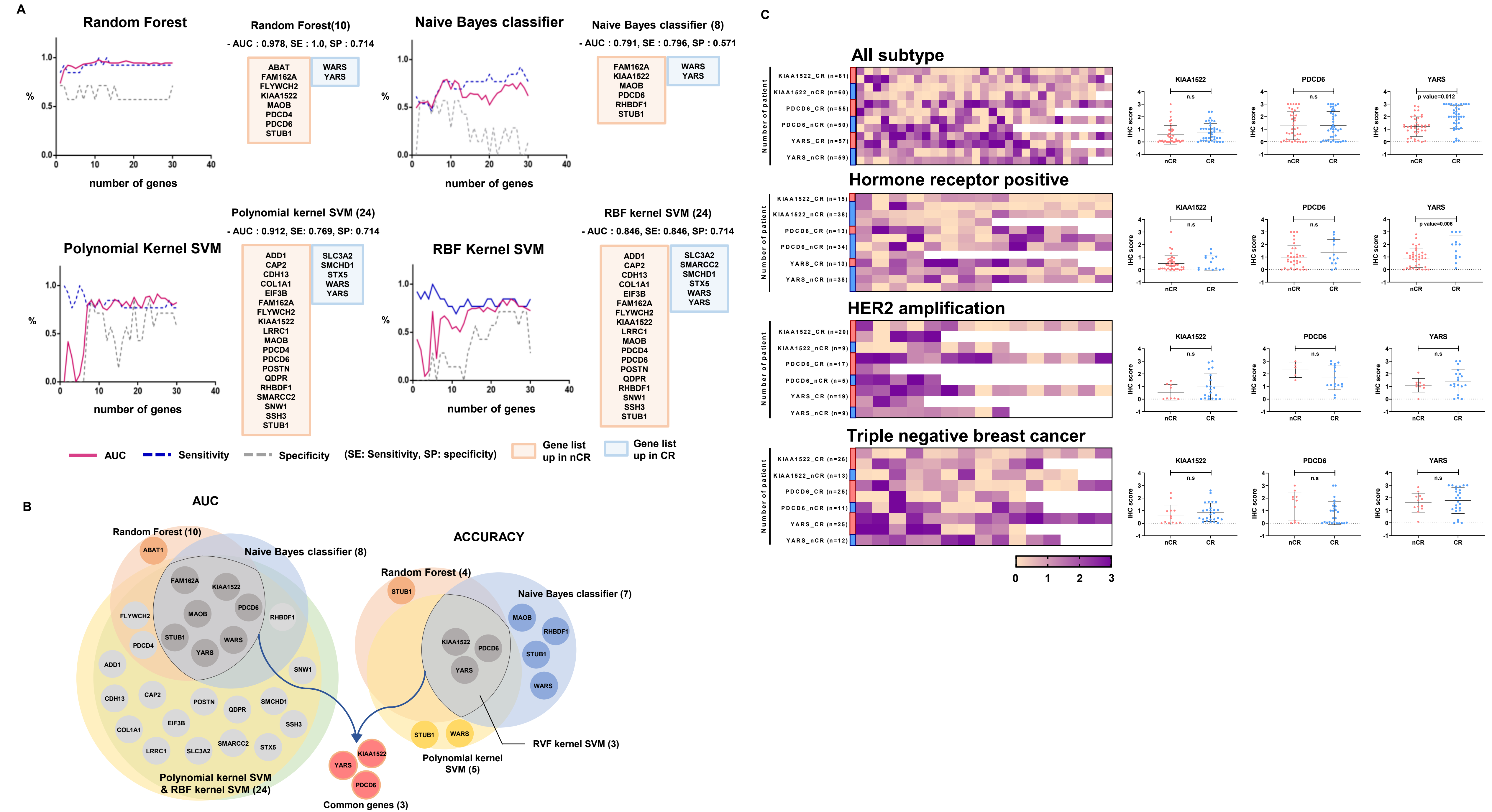
A
Random Forest
number of genes
Random Forest(10)
- AUC : 0.978, SE : 1.0, SP : 0.714
ABAT FAM162A FLYWCH2 KIAA1522 MAOB PDCD4 PDCD6 STUB1
WARS YARS
Naive Bayes classifier
Naive Bayes classifier (8)
- AUC : 0.791, SE : 0.796, SP : 0.571
FAM162A KIAA1522 MAOB PDCD6 RHBDF1 STUB1
WARS YARS
Polynomial Kernel SVM
Polynomial kernel SVM (24)
- AUC : 0.912, SE: 0.769, SP: 0.714
ADD1 CAP2 CDH13 COL1A1 EIF3B FAM162A FLYWCH2 KIAA1522 LRRC1 MAOB PDCD4 PDCD6 POSTN QDPR RHBDF1 SMARCC2 SNW1 SSH3 STUB1
SLC3A2 SMCHD1 STX5 WARS YARS
RBF Kernel SVM
RBF kernel SVM (24)
- AUC : 0.846, SE: 0.846, SP: 0.714
ADD1 CAP2 CDH13 COL1A1 EIF3B FAM162A FLYWCH2 KIAA1522 LRRC1 MAOB PDCD4 PDCD6 POSTN QDPR RHBDF1 SNW1 SSH3 STUB1
SLC3A2 SMARCC2 SMCHD1 STX5 WARS YARS
AUC
Sensitivity
Specificity
(SE: Sensitivity, SP: specificity)
Gene list up in nCR
Gene list up in CR
B
AUC
Random Forest (10)
Naive Bayes classifier (8)
Polynomial kernel SVM & RBF kernel SVM (24)
ACCURACY
Random Forest (4)
Naive Bayes classifier (7)
RVF kernel SVM (3)
Polynomial kernel SVM (5)
Common genes (3)
C
All subtype
Hormone receptor positive
HER2 amplification
Triple negative breast cancer
Number of patient
KIAA1522_CR (n=61)
KIAA1522_nCR (n=60)
PDCD6_CR (n=55)
PDCD6_nCR (n=50)
YARS_CR (n=57)
YARS_nCR (n=59)
KIAA1522_CR (n=15)
KIAA1522_nCR (n=38)
PDCD6_CR (n=13)
PDCD6_nCR (n=34)
YARS_CR (n=13)
YARS_nCR (n=38)
KIAA1522_CR (n=20)
KIAA1522_nCR (n=9)
PDCD6_CR (n=17)
PDCD6_nCR (n=5)
YARS_CR (n=19)
YARS_nCR (n=9)
KIAA1522_CR (n=26)
KIAA1522_nCR (n=13)
PDCD6_CR (n=25)
PDCD6_nCR (n=11)
YARS_CR (n=25)
YARS_nCR (n=12)
IHC score
p value=0.012
p value=0.006
n.s

Figure 3

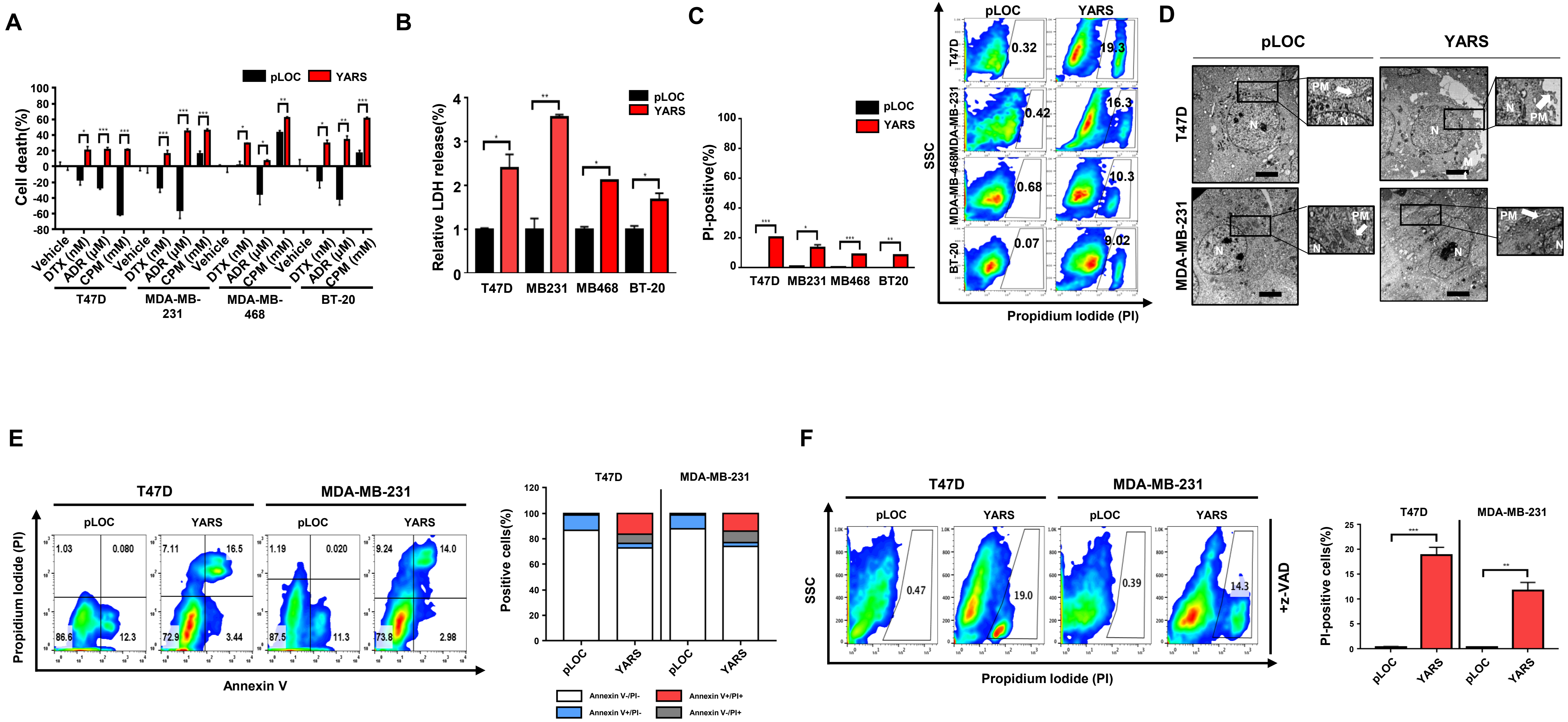

A
pLOC
YARS
Cell death(%)
Vehicle
DTX (nM)
ADR (μM)
CPM (mM)
T47D
MDA-MB-231
MDA-MB-468
BT-20
B
Relative LDH release(%)
T47D
MB231
MB468
BT-20
C
PI-positive(%)
T47D
MB231
MB468
BT20
SSC
Propidium Iodide (PI)
0.32
19.3
0.42
16.3
0.68
10.3
0.07
9.02
D
T47D
MDA-MB-231
PM
N
M
E
Propidium Iodide (PI)
Annexin V
1.03
0.080
86.6
12.3
7.11
16.5
72.9
3.44
1.19
0.020
87.5
11.3
9.24
14.0
73.8
2.98
Positive cells(%)
Annexin V-/PI-
Annexin V+/PI+
Annexin V+/PI-
Annexin V-/PI+
F
0.47
19.0
0.39
14.3
+z-VAD
PI-positive cells(%)

Figure 4

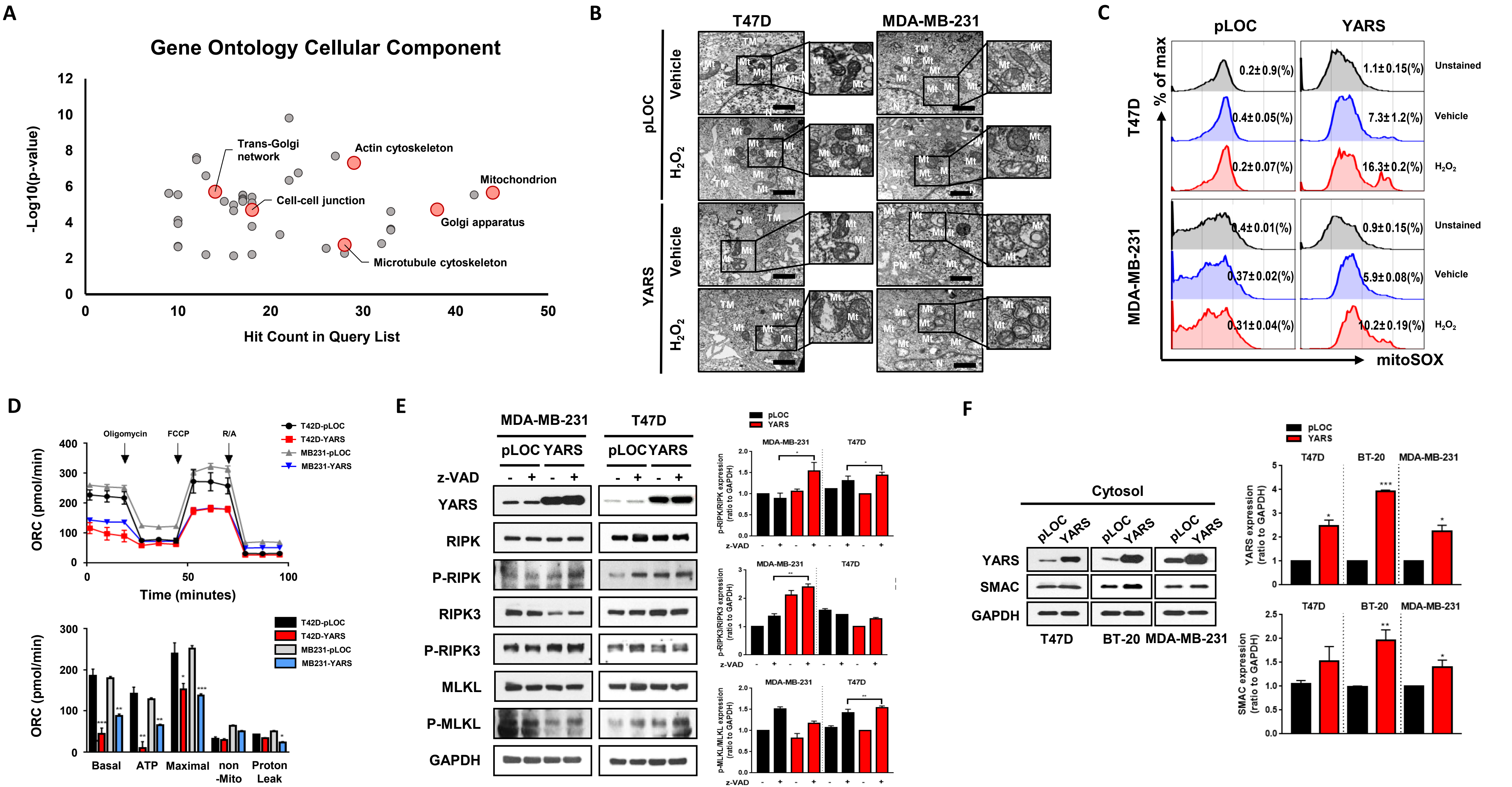
A
Gene Ontology Cellular Component
-Log10(p-value)
Hit Count in Query List
Trans-Golgi network
Actin cytoskeleton
Mitochondrion
Cell-cell junction
Golgi apparatus
Microtubule cytoskeleton
B
T47D
MDA-MB-231
pLOC
YARS
Vehicle
H2O2
C
pLOC
YARS
% of max
mitoSOX
T47D
MDA-MB-231
Unstained
Vehicle
H2O2
0.2±0.9(%)
1.1±0.15(%)
0.4±0.05(%)
7.3±1.2(%)
0.2±0.07(%)
16.3±0.2(%)
0.4±0.01(%)
0.9±0.15(%)
0.37±0.02(%)
5.9±0.08(%)
0.31±0.04(%)
10.2±0.19(%)
D
Oligomycin
FCCP
R/A
T42D-pLOC
T42D-YARS
MB231-pLOC
MB231-YARS
ORC (pmol/min)
Time (minutes)
Basal
ATP
Maximal
non -Mito
Proton Leak
E
MDA-MB-231
T47D
pLOC YARS
z-VAD
YARS
RIPK
P-RIPK
RIPK3
P-RIPK3
MLKL
P-MLKL
GAPDH
p-RIPK/RIPK expression (ratio to GAPDH)
p-RIPK3/RIPK3 expression (ratio to GAPDH)
p-MLKL/MLKL expression (ratio to GAPDH)
F
Cytosol
pLOC
YARS
SMAC
GAPDH
T47D
BT-20
MDA-MB-231
YARS expression (ratio to GAPDH)
SMAC expression (ratio to GAPDH)

Figure 5

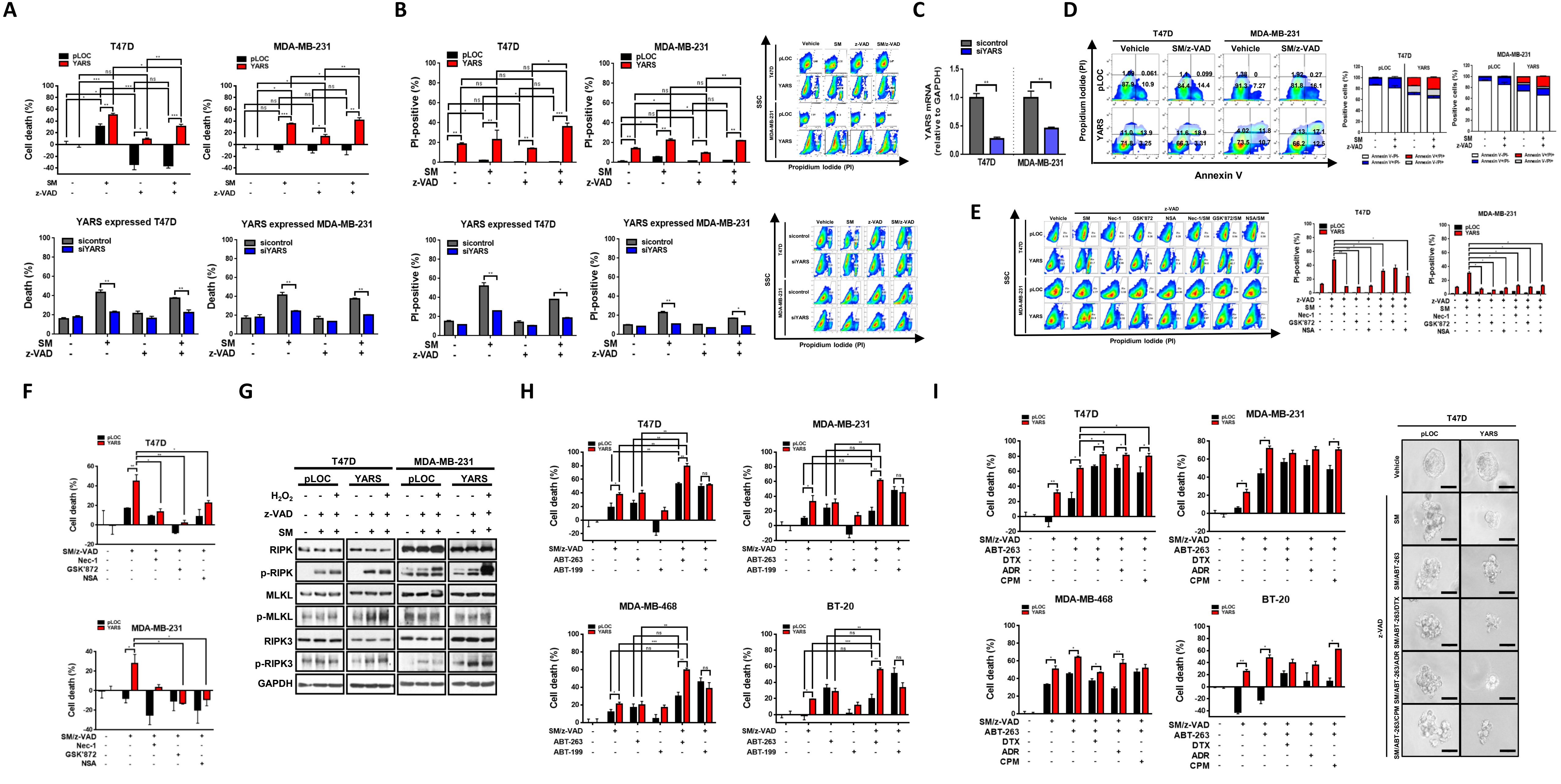
A
B
C
D
E
F
G
H
I
T47D
MDA-MB-231
YARS expressed T47D
YARS expressed MDA-MB-231
MDA-MB-468
BT-20
Propidium Iodide (PI)
Annexin V

Figure 6

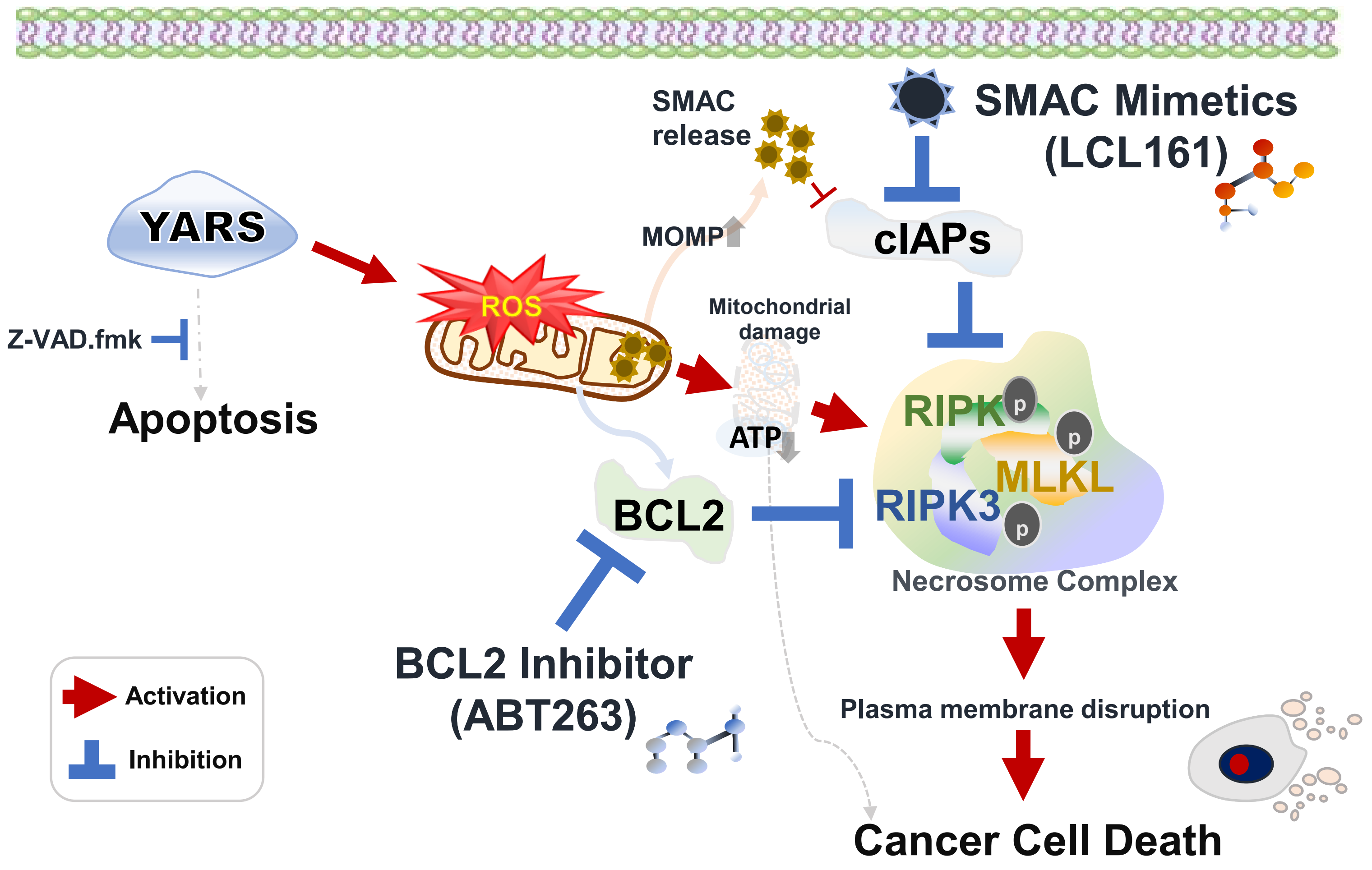

SMAC release
SMAC Mimetics
(LCL161)
YARS
MOMP
cIAPs
ROS
Mitochondrial damage
Z-VAD.fmk
Apoptosis
RIPK
MLKL
ATP
BCL2
RIPK3
Necrosome Complex
Activation
Inhibition
BCL2 Inhibitor
(ABT263)
Plasma membrane disruption
Cancer Cell Death

Supplementary Figure S1

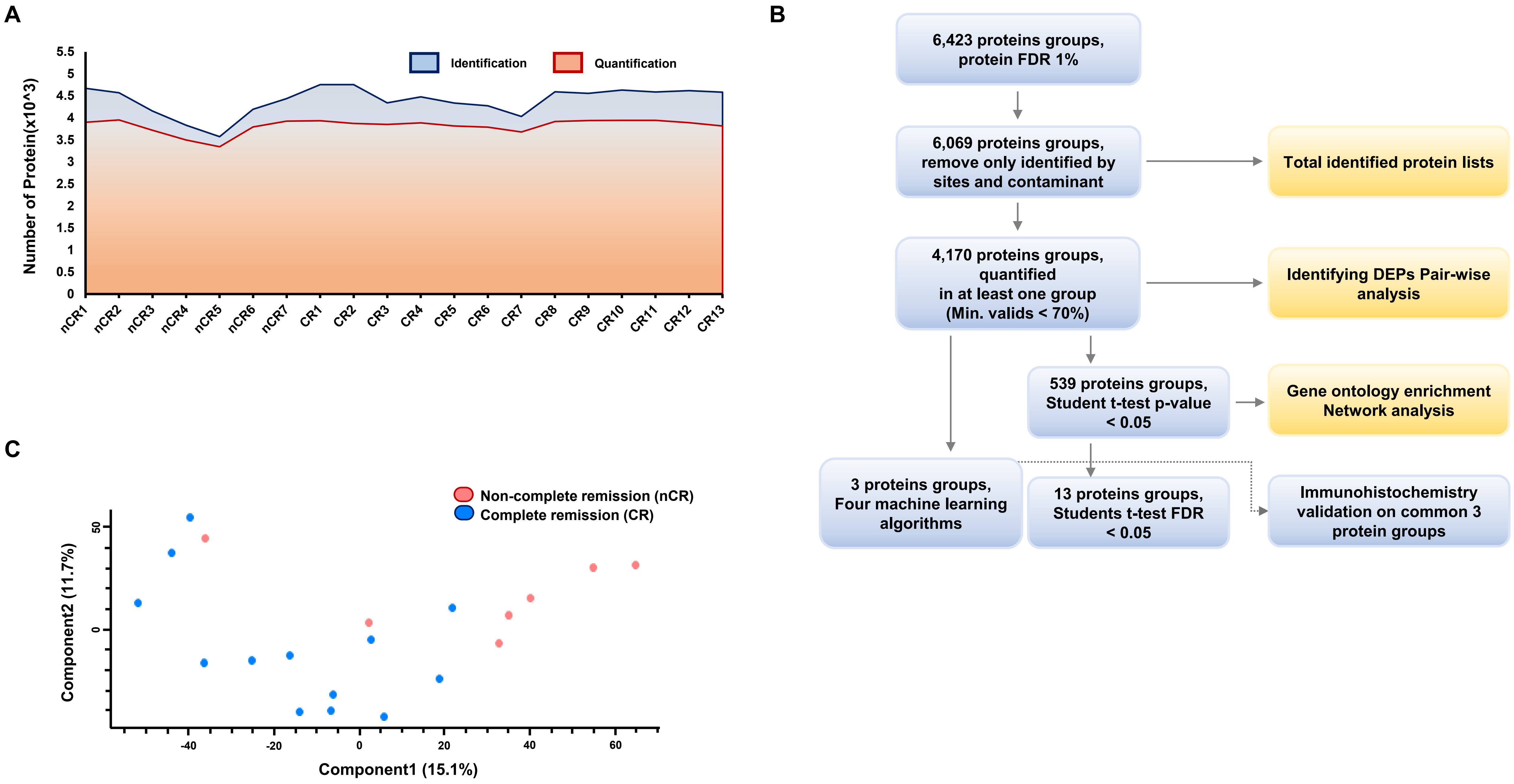

Supplementary Figure S2

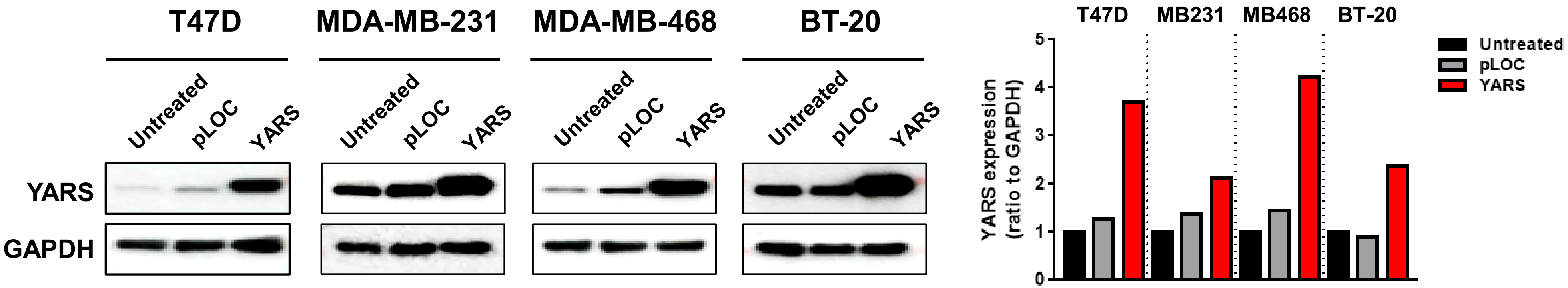

Supplementary Figure S3

A

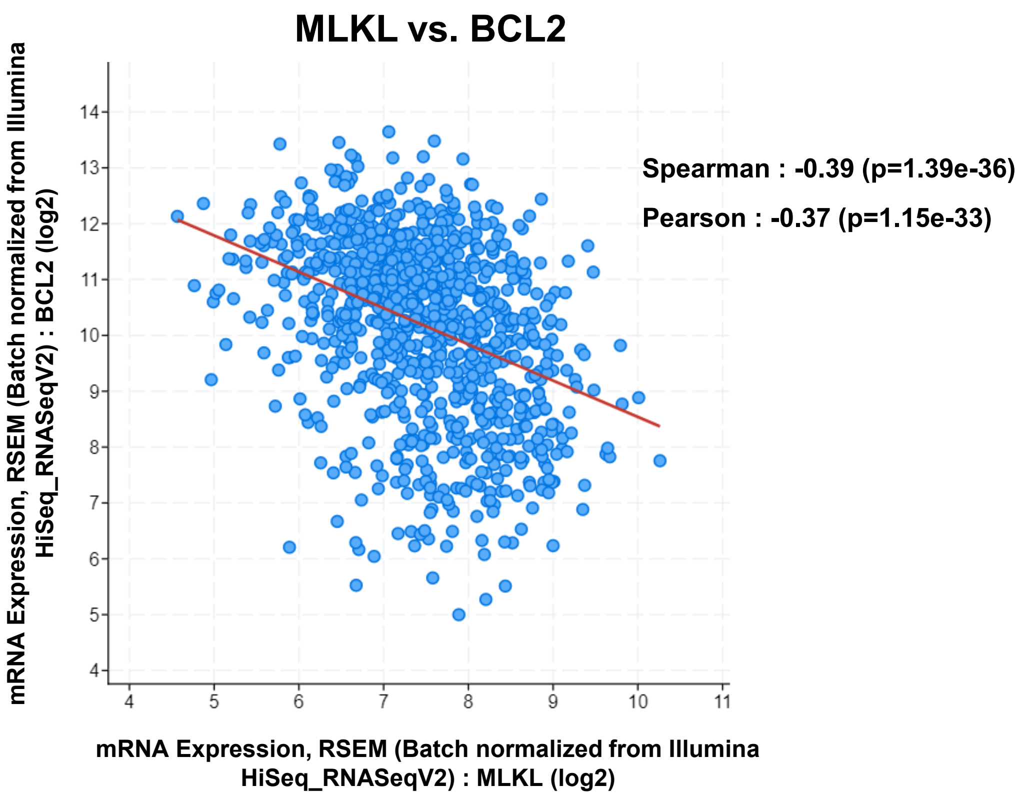

B

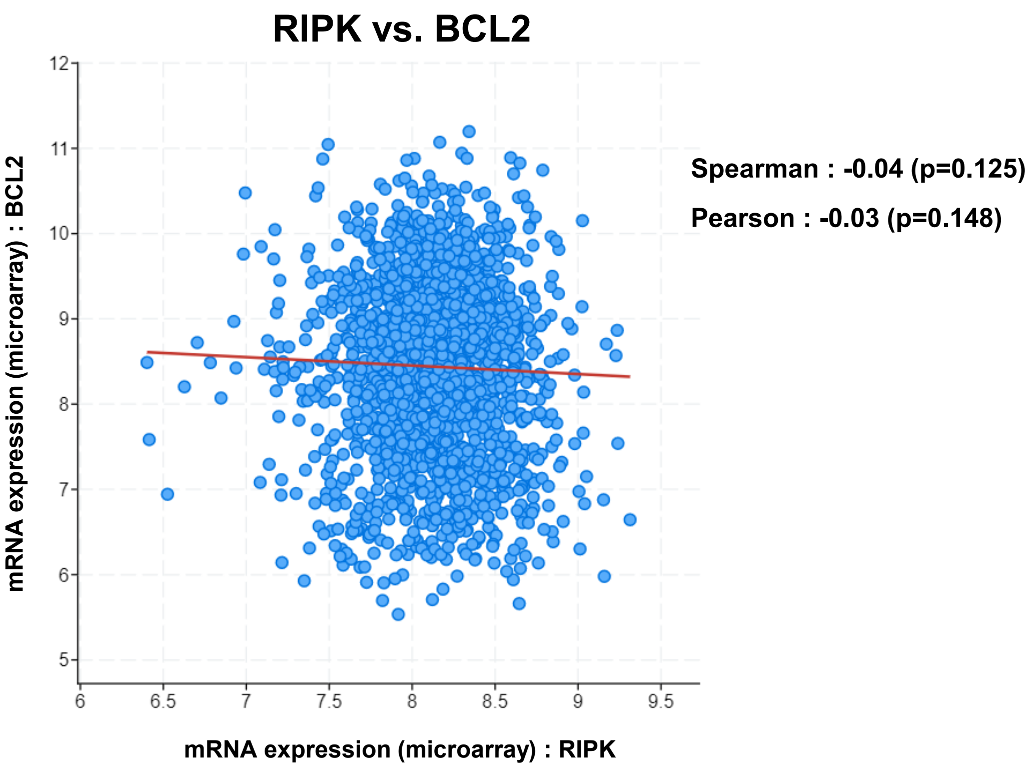

Supplementary Figure S4

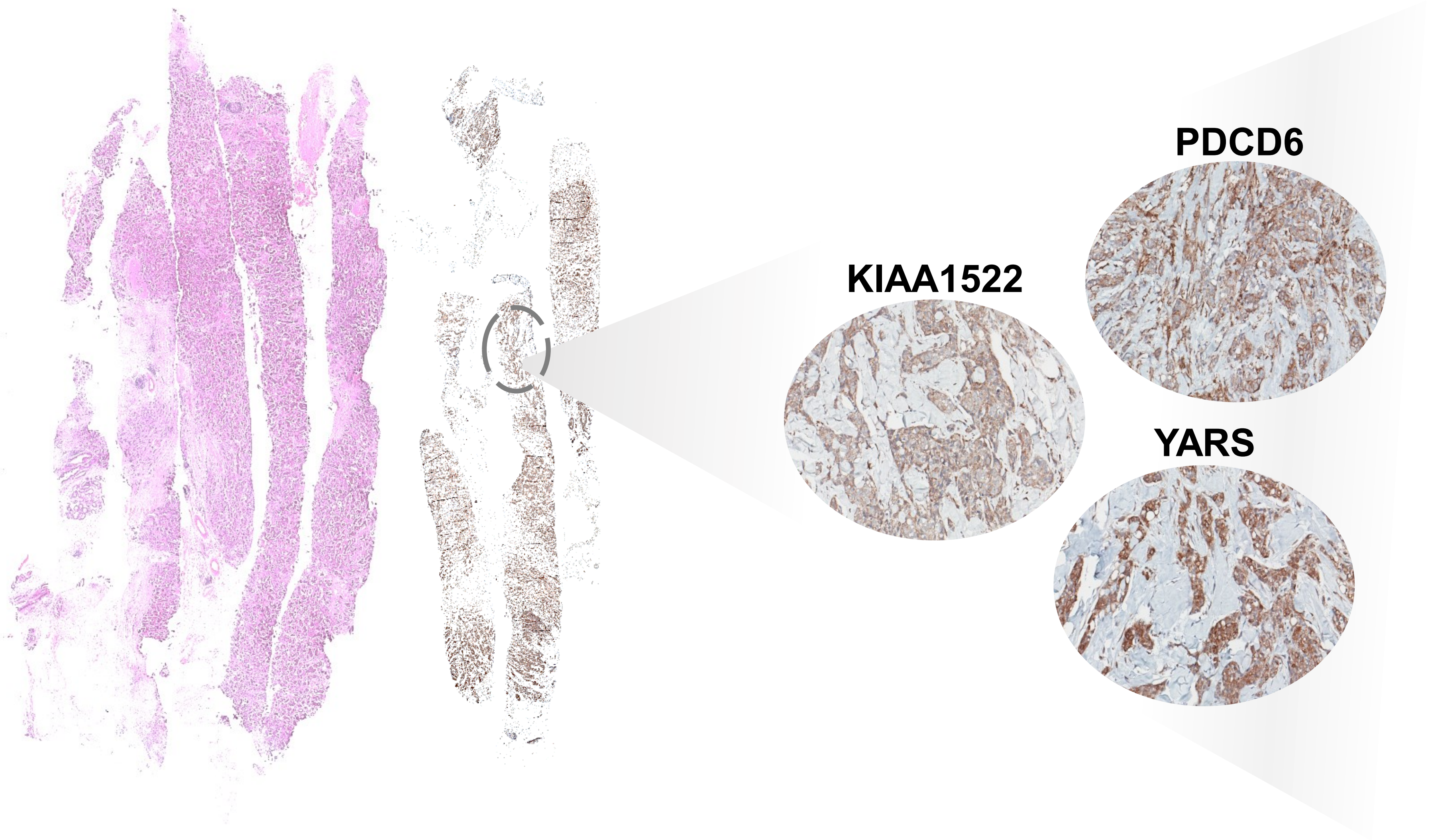

Supplementary Figure S5

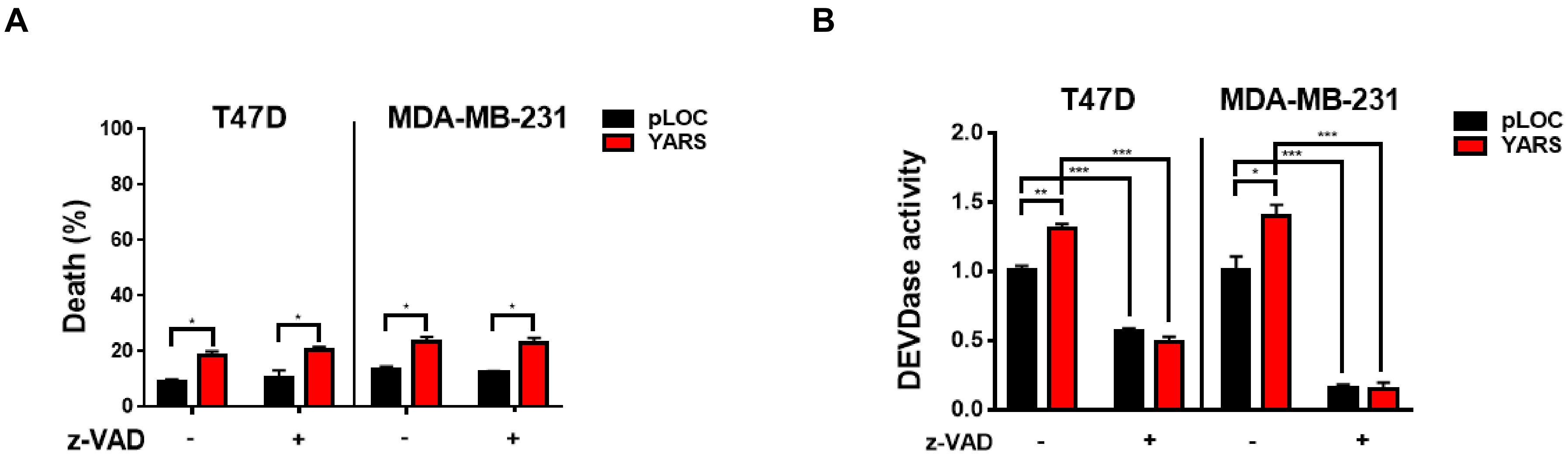

Supplementary Figure S6

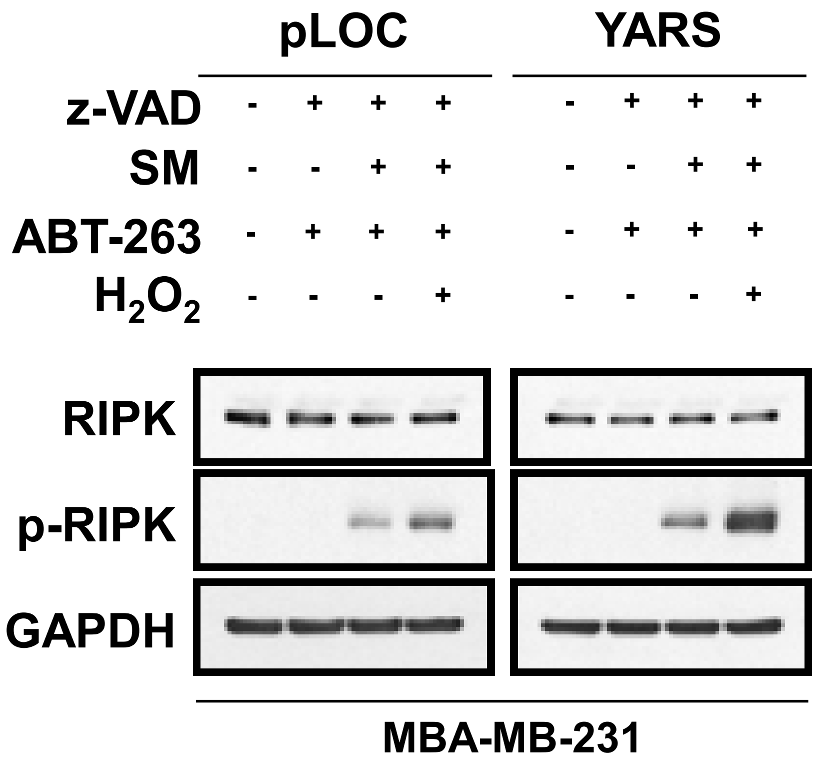

Supplement: Supplementary file 1 — Additional file 1. “BCR_revision Figure. [file 13058_2020_1367_MOESM1_ESM.pdf]
