# Supplementary material for: Combined the SMAC mimetic and BCL2 inhibitor sensitizes neoadjuvant chemotherapy by targeting necrosome complexes in tyrosine aminoacyl-tRNA synthase-positive breast cancer
Source: Breast Cancer Res. 2020 Nov 25;22:130. doi: 10.1186/s13058-020-01367-7 (PMC7687715; doi:10.1186/s13058-020-01367-7)
Supplement: Supplementary file 3 — Additional file 3. Supplementary File. [file 13058_2020_1367_MOESM3_ESM.docx]

**Supplementary Methods**

**Patient and clinical tissue sample selection**

All pathologic specimens enrolled in this study were collected from the Seoul National University Hospital biorepository operated by the Department of Pathology. Inclusion criteria for this study accepted (a) female patients who received chemotherapeutic medication with Adriamycin (ADR), cyclophosphamide (CPM) and docetaxel (DTX) as preoperative therapy [1]; (b) histologically confirmed T2 tumors with positive axillary nodes (N1); (c) biopsy samples available per-chemotherapeutic needle as well as post-chemotherapeutic surgical specimens for microscopic assessment of therapeutic effectiveness by the American Joint Committee on Cancer TNM [2]; (d) available immunohistochemical information for intrinsic subtyping. Tissue samples were divided into two sets as follows: a discovery set consisting of a total of 20 needle biopsy samples collected from 13 patients with non-pathologic complete remission (nCR) and 7 with pathologic complete remission (CR) for mass spectrometry analysis (Supplementary Table S1). Immunocytochemical validation of selected proteomic biomarkers included an independent cohort of 123 cases of needle biopsy samples, divided into 61 nCR and 62 CR cases [3]. The presence of residual tumor cells was evaluated from hematoxylin and eosin (H&E)-stained histological sections were prepared by two experienced pathologists. Moreover, the study protocol was approved by the Institutional Review Board at Seoul National University Hospital (IRB no. 1412-111-634).

**Label-free quantification and statistical analyses**

The FFPE 10 μm sections were deparaffinized in xylene for 5 min, followed by absolute ethanol twice for 3 min. Sections were then hydrated twice in 85% ethanol for 1.5 min, and distilled water for 3 min. Following vacuum-drying, the tissue samples were scraped off the slides into microfuge tubes, and extraction buffer (4% SDS; 1 mM TCEP; and 0.3 M Tris, pH 8.5) was added to each tube. Samples were briefly sonicated and incubated for 2h at 95°C. Extracted proteins were precipitated by adding chilled acetone at a volume ratio of 1:5 buffer to acetone, followed by incubation at -20°C for 16 h. Samples were washed with 200 µl of chilled acetone, and protein pellets were collected by centrifugation at 15,000 rpm for 10 min and then air-dried. Protein concentrations were measured using a bicinchoninic acid-reducing agent compatible kit (Thermo Fisher Scientific Inc., Rockford, IL, USA).

The peptide sample was prepared with 100 µg of proteins per sample and digested using the filter-aided sample preparation procedure, as described previously [4]. Eluted peptides were desalted and purified using C18 Stage Tips, as previously described [5]. Briefly, C18 Empore disk membranes (3M, Bracknell, UK) were packed into the bottom of 200 µL yellow pipette tips, followed by POROS 20 R2 reversed-phase media (Applied Biosystems, Foster City, CA, USA) which was dissolved in 1 mL MeOH. 100 µL of the mixture was loaded separately into the tip for two rounds of filtration with MeOH. Packed microcolumns were washed three times, with 100 µL of MeOH and 100% acetonitrile (ACN) consecutively and then equilibrated three times with 100 µL 0.1% TFA. Samples were loaded, and columns washed with 100 µL 0.1% TFA, and then the peptides were subsequently eluted with 100 µL of a series of elution ACN buffers in 0.1% formic acid. All eluates were dried in Speed-Vac and stored at -80°C until LC‐MS/MS analysis.

Desalted pooled peptides were fractionated using the stage-tip based high-pH peptide fractionation method [6]. Briefly, the lyophilized peptides were dissolved in 200 μL of loading buffer (15 mM ammonium hydroxide solution, pH 10, and 2% acetonitrile) then separated on a pipette based C18 microcolumn. The microcolumn was rinsed three times, with 100 μL 100% methanol and then three times with 100 μL 100% acetonitrile (ACN). The column was then conditioned with 100 μL of loading buffer using a syringe. Peptides were loaded onto the column at a pH 10. An ACN gradient of 2, 5, 7.5, 10, 12.5, 15, 17.5, 20, 22.5, 25, 27.5, 30, 32.5, 35, 40, 50, 60, 70, 80, and 100% was used to elute 20 fractions, which were collected into six tubes discontinuously to distribute eluents of varying hydrophobicity. Six fractions were lyophilized in a speed-vacuum centrifuge and stored at − 80 °C.

LC-MS/MS analysis was performed using a Q Exactive Plus Hybrid Quadrupole-Orbitrap mass spectrometer (Thermo Fisher Scientific Inc.), coupled to an Ultimate 3000 RSLC system (Dionex, Sunnyvale, CA, USA) via a nanoelectrospray source, as previously described [6, 7]. Peptide samples were separated on a two-column system, consisting of a trap column and an analytic column (75 μm × 50 cm) with a 120 min gradient from 7% to 32% acetonitrile at 300 nl/min and analyzed by mass spectrometry. Survey scans (350 to 1650 m/z) were acquired with a resolution of 70,000 at m/z 200. MS/MS spectra were acquired at an HCD-normalized collision energy of 30 with a resolution of 17,500, at m/z 200. The maximum ion injection times for the full scan and MS/MS scan were 20 and 100 ms, respectively.

Mass spectra were processed using a MaxQuant version 1.5.3.1 [8]. MS/MS spectra were searched by utilizing the Human UniProt protein sequence database (December 2014, 88,657 entries) using the Andromeda search engine with a 6-ppm precursor ion tolerance for total protein level analysis [9]. Primary searches were performed using the MS/MS ion tolerance and set to 20 ppm. Cysteine carbamidomethylation N-acetylation of protein and oxidation of methionine were set as fixed and variable modifications, respectively. Enzyme specificity was set to full tryptic digestion. Peptides with a minimum length of six amino-acids and up to two missed cleavages were considered. The required false discovery rate (FDR) was set to 1% at the peptide, protein, and modification level. We enabled the ‘Match between Runs’ option on the MaxQuant platform to maximize the number of quantification events across samples.

**Supplementary References**

1 von Minckwitz G, Raab G, Caputo A, Schutte M, Hilfrich F, Blohmer JU *et al*. Doxorubicin with cyclophosphamide followed by docetaxel every 21 days compared with doxorubicin and docetaxel every 14 days as preoperative treatment in operable breast cancer: The GEPARDUO Study of the German Breast Group. *Journal of Clinical Oncology* 2005; 23: 2676-2685.

2 Edge SB, Compton CC. The American Joint Committee on Cancer: the 7th edition of the AJCC cancer staging manual and the future of TNM. *Ann Surg Oncol* 2010; 17: 1471-1474.

3 von Minckwitz G, Untch M, Blohmer JU, Costa SD, Eidtmann H, Fasching PA *et al*. Definition and Impact of Pathologic Complete Response on Prognosis After Neoadjuvant Chemotherapy in Various Intrinsic Breast Cancer Subtypes. *Journal of Clinical Oncology* 2012; 30: 1796-1804.

4 Wisniewski JR, Zougman A, Nagaraj N, Mann M. Universal sample preparation method for proteome analysis. *Nat Methods* 2009; 6: 359-362.

5 Rappsilber J, Mann M, Ishihama Y. Protocol for micro-purification, enrichment, pre-fractionation and storage of peptides for proteomics using StageTips. *Nat Protoc* 2007; 2: 1896-1906.

6 Han D, Jin J, Woo J, Min H, Kim Y. Proteomic analysis of mouse astrocytes and their secretome by a combination of FASP and StageTip-based, high pH, reversed-phase fractionation. *Proteomics* 2014; 14: 1604-1609.

7 Han D, Moon S, Kim Y, Kim J, Jin J, Kim Y. In-depth proteomic analysis of mouse microglia using a combination of FASP and StageTip-based, high pH, reversed-phase fractionation. *Proteomics* 2013; 13: 2984-2988.

8 Tyanova S, Temu T, Cox J. The MaxQuant computational platform for mass spectrometry-based shotgun proteomics. *Nat Protoc* 2016; 11: 2301-2319.

9 Cox J, Neuhauser N, Michalski A, Scheltema RA, Olsen JV, Mann M. Andromeda: a peptide search engine integrated into the MaxQuant environment. *J Proteome Res* 2011; 10: 1794-1805.
